# Interspecific common bean population derived from *Phaseolus acutifolius* using a bridging genotype demonstrate useful adaptation to heat tolerance

**DOI:** 10.3389/fpls.2023.1145858

**Published:** 2023-05-12

**Authors:** Sergio Cruz, Juan Lobatón, Milan O. Urban, Daniel Ariza-Suarez, Bodo Raatz, Johan Aparicio, Gloria Mosquera, Stephen Beebe

**Affiliations:** Bean Breeding Program, International Center for Tropical Agriculture (CIAT), Palmira, Colombia

**Keywords:** genome wide association study (GWAS), phaseolus acutifolius (tepary bean), introgression analysis, interspecific, heat tolerance, yield

## Abstract

Common bean (*Phaseolus vulgaris* L.) is an important legume crop worldwide and is a major nutrient source in the tropics. Common bean reproductive development is strongly affected by heat stress, particularly overnight temperatures above 20°C. The desert Tepary bean (*Phaseolus acutifolius* A. Gray) offers a promising source of adaptative genes due to its natural acclimation to arid conditions. Hybridization between both species is challenging, requiring *in vitro* embryo rescue and multiple backcrossing cycles to restore fertility. This labor-intensive process constrains developing mapping populations necessary for studying heat tolerance. Here we show the development of an interspecific mapping population using a novel technique based on a bridging genotype derived from *P. vulgaris*, *P. Acutifolius* and *P. parvifolius* named VAP1 and is compatible with both common and tepary bean. The population was based on two wild *P. acutifolius* accessions, repeatedly crossed with Mesoamerican elite common bush bean breeding lines. The population was genotyped through genotyping-by-sequencing and evaluated for heat tolerance by genome-wide association studies. We found that the population harbored 59.8% introgressions from wild tepary, but also genetic regions from *Phaseolus parvifolius*, a relative represented in some early bridging crosses. We found 27 significative quantitative trait loci, nine located inside tepary introgressed segments exhibiting allelic effects that reduced seed weight, and increased the number of empty pods, seeds per pod, stem production and yield under high temperature conditions. Our results demonstrate that the bridging genotype VAP1 can intercross common bean with tepary bean and positively influence the physiology of derived interspecific lines, which displayed useful variance for heat tolerance.

## Introduction

Common bean (*Phaseolus vulgaris* L.) is the most widely consumed legume in Latin America and Africa. Its seeds are of nutritional interest due to their taste and beneficial nutritional profile. In some contexts it provides up to one third of daily protein intake (Beebe, 2012). Living populations of common bean’s wild ancestors have been found in an extensive tropical and subtropical area ranging from northern Mexico (approx. 30 °N.) to northwestern Argentina (approx. 35 °S; (Gepts, 1998). Generally sub-humid forest clearings are characterized by well-drained soils and bimodal rainfall patterns with a short dry period between the two wet seasons. Evolving in these conditions, the common bean’s wild ancestor was rarely exposed to extreme soil constraints, high temperature or long drought conditions, and thus its modern common bean descendant is sensitive to such constraints (Toro et al., 1990; Gaut, 2014). Wild common bean is organized in two geographically isolated and genetically differentiated wild genepools (Mesoamerican and Andean) that diverged from a common ancestral form ~165.000 years ago and from these wild genepools approximately 8.000 years ago common bean was domesticated in Mexico and South America (Schmutz et al., 2014).

Reproductive development of domesticated common bean is especially susceptible to high temperature stress with day and night temperatures greater than 30°C and 20°C, respectively, resulting in significant yield reduction (Porch & Jahn, 2001). Published studies suggest that heat sensitivity is caused by damage during reproductive development of male structures compromising pollen grain viability and modifying normal pollen tube development within the style, which impacts all yield components (Rainey & Griffiths, 2005). Additionally, it has been reported that heat stress disrupts translocation between sources and sinks, thus limiting flowering and seed filling (Soltani et al., 2020). Common bean is vulnerable to expected future climate scenarios especially for increases in average ambient temperatures above the range of bean adaptation (Beebe et al., 2011; Hummel et al., 2018).

The tepary bean (*Phaseolus acutifolius*) is linked to the tertiary common bean genepool. However, it evolved in the hot, arid Mexican and southwestern US deserts (Freytag & Debouck, 2002). It exhibits multiple traits associated with drought and heat resistance, this being a promising source of useful genes to improve the genetics of common bean. These include i) tolerance to temperatures greater than 32°C, ii) stomatal control, iii) dehydration avoidance, iv) excellent photo-assimilate mobilization to seeds, and v) a fine root system that allows it to rapidly penetrate the soil (Beebe, 2012). There is considerable genetic distance between tepary and common bean species. Thus, interspecific offspring are usually not viable and hybrid embryos abort within the mother plant pods. *In vitro* embryo rescue can increase hybrid F_1_ plants’ survival rate that are self-incompatible, so multiple backcrossing with common bean is needed to restore fertility (Honma, 1956; Garvin et al., 1997). A drawback of recurrent backcrossing with common bean is the rapid dilution of tepary introgressions. An alternative method named congruity backcrossing, which alternates *P. vulgaris* and *P. acutifolius* as the backcross parent can boost recombinations between the species (Mejía et al., 1994). Segregation distortion in interspecific populations is characterized by deviating Mendelian ratios for certain markers, which displays homozygote deficiency for the tepary allele, and thus indicating a limited recombination between both species (Garvin et al., 1997).

Multiple interspecific populations combining tepary and common bean have been developed using *in vitro* embryo rescue possessing useful variance in common bacterial blight resistance (*Xanthomonas campestris*), bruchid resistance, and cold-and drought tolerance (Honma, 1956; Kusolwa & Myers, 2008; Martinez, 2010; Souter et al., 2017; Suárez et al., 2020). These populations shared a reduced population size for early generations. This was probably due to the low *in vitro* embryo rescue success rate that constrained establishing genetic mapping populations indispensable for studying the genetic basis of complex traits such as heat resistance. However, repeated intercrossing between the species *P. acutifolius, P. parvifolius* and *P. vulgaris* at CIAT has established VAP lines that permit hybridizing common and tepary beans without using embryo rescue techniques (Barrera et al., 2022). This bridging genotype approach enables establishing sufficiently large genetic mapping populations. The research objectives reported here are: (1) developing an interspecific population combining wild tepary bean and Mesoamerican common bean by using the bridging genotype VAP1, (2) characterizing the interspecific population in high temperatures using heated greenhouse environments, (3) assessing the introgression levels in the population, and (4) evaluating the association between introgression fragments and the population’s phenotypic responses under controlled and high temperature conditions.

## Materials and methods

### Plant material

**Figure 1:**
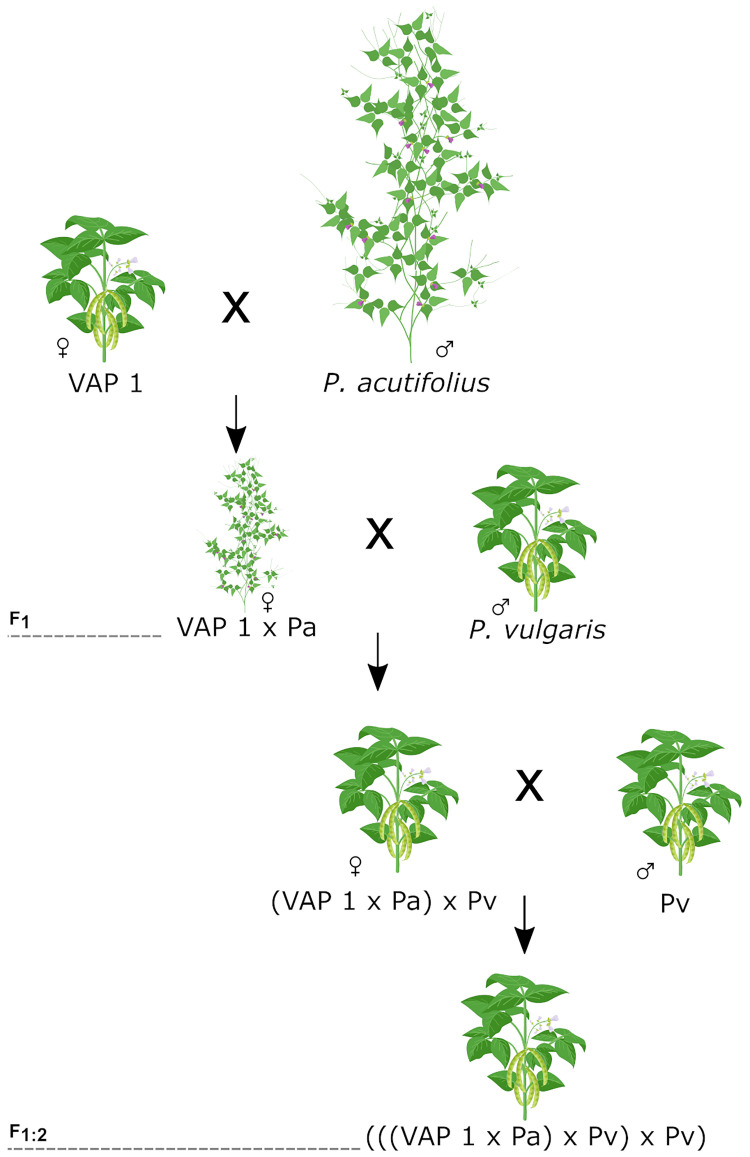
Crossing scheme used for IMAWT population development. First the bridging genotype (VAP 1) and a wild tepary accession was intercrossed using the first as a female. The F_1_ was then crossed twice with a common bean parental line to produce the secondary F_1:2_ generation.

We developed a unique Interspecific Mesoamerican X Wild Tepary (IMAWT) population with the following crossing scheme: ((*P. acutifolius* x VAP1) x *P. vulgaris*) x *P. vulgaris*). The bridging line VAP1 (with parentage of *P. vulgaris*, *P. acutifolius*, and *P. parvifolius*) allowed us to create interspecific crosses without embryo rescue (Barrera et al., 2022). We obtained F_1_ plants crossing VAP1 with two wild accessions of *P. acutifolius* (G40056 or G40287) followed by two crosses to P*. vulgaris* (**Figure 1**). The common bean parental lines correspond to five elite breeding lines from the Mesoamerican genepool (SMR155, SEF10, SMC214, ICTA Ligero, and SEN118). They are drought tolerant and represent different commercial grain classes. The line SEF10 is an accession that also presents tepary crosses in its pedigree. We obtained 50 F_1:2_ genotypes from 14 different combinations of the above-mentioned parental lines. The accessions’ seed stocks were grown on until generation F_5_ *via* single-seed descent by selecting random plants in optimal field conditions to avoid biased selection of certain plant idiotypes. The F_5_ population embraced 892 lines. In 2019 we performed a non-replicated screening trial in two greenhouses with propane heaters that maintained the temperature above 24 °C for the entire cultivation cycle (**Supplementary Figure 1**). The plot contained four sister plants planted in soil, with 5 cm between plants, and 40 cm between plots for a total of 408 experimental units (EUs) per greenhouse. We visually estimated the number of pods per plot in two greenhouses at the International Center for Tropical Agriculture (CIAT), Palmira, Valle del Cauca (03°30’ 20.39” N, 76° 20’ 28.13” W, 973.22 m.a.s.l). We selected 302 F_5_ population representatives. Two lines with the best pod load were selected from the F_2:3_ groups and one line with the worst was selected from the F_1:2_ groups to guarantee a wider range of phenotypic variation. We increased those 302 F_5_ introgression lines (ILs) by bulking to create the F_5:6_ IMAWT population (**Supplementary Table 1**).

### Heat tolerance trial

The IMAWT population was evaluated at CIAT, Palmira in three environments: two in greenhouses with controlled temperature (GH1 and GH2) named jointly as heat stress environments (HS); and one in an open field trial without any heat stress (Non-stress environment, NS). In greenhouses, propane heaters maintained a minimum air temperature of 25°C during the growing cycle. In both HS and NS environments, drip irrigation was used to maintain soil water content at field capacity until necessary. The irrigation schemes were identical for GH1 and GH2. Fertilizer and pesticide applications did not differ between environments. Generally, all standard operation procedures were similar within environments. The main climatic parameters that logically differed between HS and NS environments were air temperature and photosynthetic active radiation (PAR). During the entire cultivation period (from sowing until final harvest), the minimum, maximum and average air temperatures were consistently higher in HS environments than in NS (**Supplementary Figure 2A**). Average day/night air temperatures were 32/25 °C and 33/25 °C in GH1 and GH2, respectively; and 30/22 °C in NS. The minimum and maximum temperatures are consistently similar within HS and follow the climate dynamics observable in NS. The temperature in both GHs never dropped below 25 °C. Similarly, average relative air humidity was higher in HS environments (with some short exceptions; 81.7% and 80% for GH1 and GH2, respectively) than in the NS environment (75%; **Supplementary Figure 2B**). PAR accumulated by day was higher in NS (48 mol/m^2^day) than in HS environments (25.2 and 25.4 mol/m^2^day for GH1 and GH2, respectively) over the entire crop cycle (**Supplementary Figure 2C**).

Our partially replicated experimental design used 30% of the population with a second replicate in each environment (Moehring et al., 2014). Each EU contained four plants, establishing 408 EU in each environment. Plants were harvested when they reached senescence, at around 95 days after sowing (DAS) in HS environments and 75 DAS in NS. The number of harvested plants per plot was recorded, and plants were dried at 70°C until constant moisture content. The number of pods per plant (PP), number of seeds per plant (SP), the weight of 100 seeds (SW), dry weight of leafless stems per plot (StWP), number of empty pods per plant (EPP), yield per plant (YdPl), average number of seeds per pod (NSP = SP/PP), harvest index (HI = seed weight per plant/(pod weight per plant + StWP)) and pod harvest index (PHI) were registered/calculated following the methodology proposed by Assefa et al. (2017).

We fitted a mixed linear model for each environment, including the rows and columns as a random effect. This model was fitted using the statistical software Mr. Bean, which is based on the ‘SpATS’ R package (Rodríguez et al., 2018; Aparicio et al., 2019). The genotype was included as a random factor to calculate the Best Linear Unbiased Predictors (BLUPs) and broad sense Cullis heritability (Oakey et al., 2006).

### Library construction and sequencing

For DNA extraction, we sampled two 5cm-long axillary buds per plant per line at 25 DAS under field conditions using the standard CTAB method (ionic detergent cetyltrimethylammonium bromide) (Doyle & Doyle, 1987). DNA from each sample was quantified by electrophoresis using different DNA λ as concentration standards and diluted to a concentration of 100 ng/µl for all samples using ultrapure water as solvent. For DNA sequencing, we used genotyping by sequencing (GBS) using the *Ape*K1 restriction enzyme (Elshire et al., 2011). Four libraries were constructed and sequenced on the Illumina HiSeq X platform with 150 bp pair-end reads at the Macrogen facility in Seoul, South Korea (https://dna.macrogen.com/).

### Variant calling and population structure

GBS reads were de-multiplexed using the Stacks software (v 2.52) using the module *process_readtags* for paired reads (Rochette et al., 2019). Trimmomatic software (v 0.36) was used to remove adapters and low-quality bases (Bolger et al., 2014). Processed reads were mapped to *P. vulgaris* G19833 v 2.1 reference genome (Schmutz et al., 2014) using Bowtie2 software with default parameters (Langmead & Salzberg, 2012). The first variant calling per individual was performed with NGSEP software v 4.4.1 (Tello et al., 2019), considering a minimum average quality per read > 40 and a minimum depth of 3 reads. Subsequently, Bcftools (v 1.9) software was used to perform the second variant calling for the population (Li, 2011). The filters used removed variants in the reference genome repetitive zones (Lobaton et al., 2018), selecting only bi-allelic (SNPs), heterozygosity per marker ≤ 10%, and minor allele frequency (MAF) ≥ 2.5%. The genotypic matrix was set to 20% missing data by removing variants with less than 208 genotyped individuals. We included for population structure analysis the WGS samples VAP1 and *P. parvifolius* G40264 from Barrera et al. (2022).

### Introgression analysis

For introgression analysis, we selected the SNPs where the common bean parental lines (ICTA Ligero, SEN 118, SEF 10, SMR 155, and SMC 214) presented no missing data and were monomorphic. From those, we selected the variants where the wild tepary parental lines (G40056 and G40287) and G40264 (jointly named *Acutifolii* samples hereafter) present no missing data, were homozygous, and doesn’t share any allele with initially common bean parental monomorphic variants (**Supplementary Figure 3**). This set of contrasting SNPs was recoded into tree states: A (common bean origin), B (*Acutifolii* origin), or H (heterozygous), and the crossing-over points between both backgrounds were detected using SNPBinner using an emission probability of 0.99 (representing the predicted region’s genotypic homogeneity) and a minimum introgression size of 0.1% of chromosome size (Gonda et al., 2019).

### Genome-wide association analysis

Genome Wide Analysis Studies (GWAS) were performed with the method named Fixed and random model Circulating Probability Unification or FarmCPU (X. Liu et al., 2016) implemented in the R statistical package GAPIT v 3.0 (Lipka et al., 2012). We removed the wild tepary parental lines from the analysis due to their contrasting genotypic and phenotypic differences in relation to the population that could produce spurious associations. To determine the significance threshold, we used the Bonferroni threshold with an α level of 5%. Manhattan plots and QQ-plots were plotted with a custom Python script.

## Results

### Population development

We developed the IMAWT population by intercrossing two wild accessions of tepary bean (G40056 and G40287) with five Mesoamerican elite breeding lines of common bean (SEN118, ICTA Ligero, SMR155, SMC214 and SEF10). We obtained 302 F_5:6_ introgression lines from 14 parental combinations. The IMAWT population exhibited morphological characteristics normally associated with wild tepary beans such as lanceolate trifoliate leaves and angular seeds (**Supplementary Figure 4**). A total of 24.205 bi-allelic SNPs were called for 244 samples. An introgression analysis was performed to identify the segments introgressed from the sister’s species. Additionally, we performed a GWAS analysis to identify heat-tolerance related quantitative trait nucleotides (QTNs).

### Heat stress trial

**Figure 2:**
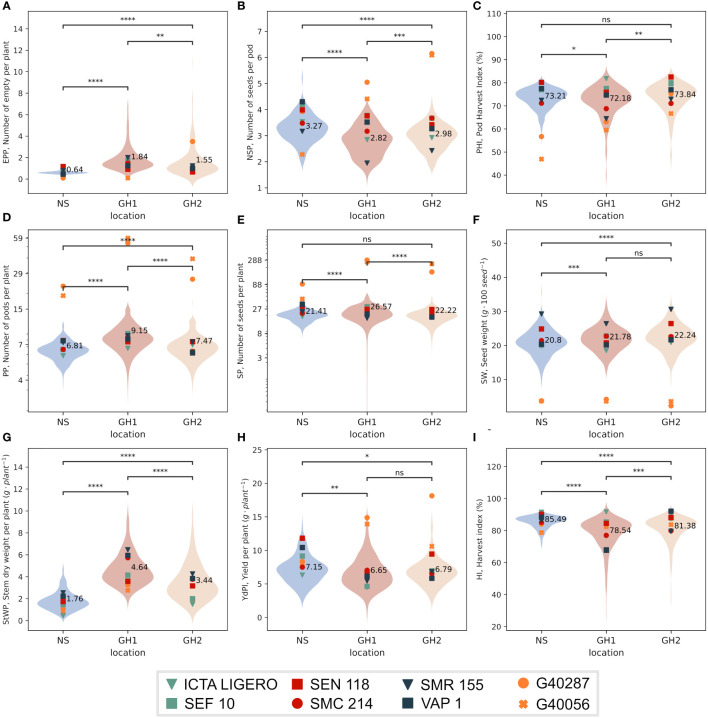
Distribution of the phenotypic values by environment in three locations; NS (Field, non-stress) and heat greenhouses GH1 and GH2. Horizontal lines represent comparisons of means using a paired t-test. The average value for environment is annotated. Markers indicate phenotypic values for the IMAWT population parents. **(A)** Empty pods per plant (EPP). **(B)** Number of seeds per pod (NSP). **(C)** Pod harvest index (PHI). **(D)** Pods per plant (PP). **(E)** Seeds per plant (SP). **(F)** Seed weight (SW). **(G)** Dry stem weight per plant (StWP). **(H)** Yield per plant (YdPl). **(I)** Harvest index (HI). Plots D and E are in log scale for better representation. Asterisks between violins represents the significance of comparison of means through independent t-test method, ns P > 0.05; * P ≤ 0.05; ** P ≤ 0.01; *** P ≤ 0.001; **** P ≤ 0.0001.

Traits evaluated at harvest differed significantly between NS and HS environments, except for PHI and SP in GH2; **Figures 2C, E**). EPP and StWP increased in HS environments relative to NS. Average StWP values in NS were 1.76 g/plant, however in GH1 and GH2 we observed values of 4.63 and 3.44 g/plant, respectively. Similarly, the overall EPP values in NS were 0.64 empty pods/plant but for GH1 and GH2 we observed 1.84 and 1.55 empty pods/plant, respectively (**Figures 2A, G**).

For YdPl, NSP, and HI traits, the average response was significantly higher in NS than in HS environments. Average YdPl in NS was 7.16 g/plant but 6.66 and 6.79 g/plant for GH1 and GH2, respectively, observing a reduction of 7% and 5% to NS, respectively (**Figure 2H**). For NSP, average value in NS was 3.27 seeds/pod, whereas we observed 2.82 and 2.98 seeds/pod in GH1 and GH2, respectively, observing an average reduction of 14% and 9% to NS, respectively (**Figure 2B**). In regards to HI the average value in NS was 85.5% whereas for GH1 and GH2 78.6 and 81.4%, observing a reduction of 7% and 5% to NS, respectively (**Figure 2I**).

For PP, SP, and SW the averages in HS environments were significantly higher than in NS. Average PP in NS was 6.81 pods/plant whereas for GH1 and GH2 was 9.15 and 7.47 pods/plant, respectively, indicating an significant increase of 34% and 9.7% (**Figure 2D**). For SP, the average in NS was 21.4 seeds/plant whereas in GH1 and GH2 it was 26.6 and 22.2 seeds/plant, respectively, observing an significant increase of 24% and 3.7% to NS (**Figure 2E**). For SW the average in NS was 20.8 g/100 seeds whereas for GH1 and GH2 it was 21.8 and 22.2 g/100 seeds observing an significant increase of 4.6% and 6.8%, respectively (**Figure 2F**).

We observed positive and significant trait correlations between HS (GH1 and GH2) environments. All correlations between GH1/GH2 are higher than for HS vs NS with an exception for SP (**Supplementary Figure 5**). Interestingly, EPP in NS did not correlate with both HS treatments. GH2 showed lower, however still significant correlations to NS for traits as PP, SP, and YdPl. The broad-sense heritabilities (H^2^) were greater than 50% for all traits except for EPP and PP in NS and StWP in the GH1. Importantly, except for StWP and SW, HS environments presented consistently higher H^2^ values than those of NS. The GH2 (except for NSP, SP and HI) presented higher values of H^2^ than GH1 (**Supplementary Table 2**).

**Figure 3:**
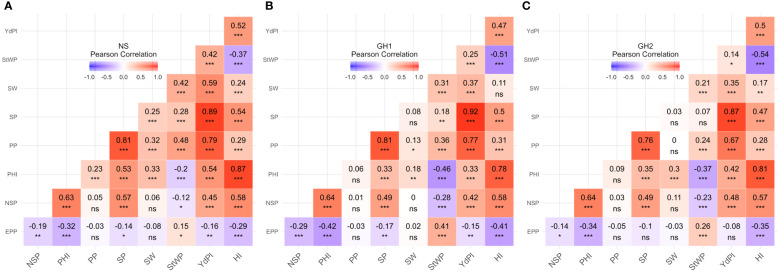
Phenotypic correlations between traits by environment. **(A)** Field. **(B)** GH1. **(C)** GH2. EPP, Empty pods per plant; NSP, Number of seeds per pod; PHI, Pod harvest index; PP, Pods per plant; SP, Seeds per plant; SW, Seed weight; StWP, Dry stem weight per plant; YdPl, Yield per plant. Asterisks indicate the significance of Pearson correlation coefficients ns P > 0.05; * P ≤ 0.05; ** P ≤ 0.01; *** P ≤ 0.001.

For each trait and each environment, the Pearson correlation coefficients were calculated. Traits correlated in a similar fashion independently of environment. However, in HS environments the magnitude of correlations was often lower than in NS (**Figure 3**). Correlations between YdPl and its components (PP, SP, SW, and NSP) were positive and significant for NS and HS, however in HS environments correlation between SW and YdPl were lower than in NS although still significant (0.37^***^ and 0.35^***^ for GH1 and GH2, respectively; and 0.59^***^ for NS). Similarly, SW correlated differentially with SP and PP (**Figure 3**). The correlation between StWP and YdPl in NS was 0.42^***^ but in HS environments was 0.25^***^ and 0.14^*^ for GH1 and GH2, respectively; **Figure 3**).

Wild tepary parental lines performed generally much better than common bean parental lines under the HS environments. Excepting for G40056 in GH2 where YdPl didn’t differ to SEN118 (**Supplementary Table 3**), we observed significant differences between tepary and common bean parental lines in HS environments supporting the idea of heat resistance traits in tepary accessions. The same negative result is visible when comparing yields from NS, where both tepary accessions (according to expectations) had very low yields. Importantly, some interspecific lines outperformed in YdPl common bean parental lines in HS environments. In GH1 we observed 107 interspecific lines with a higher YdPl than SMC214 (7.04 g/plant), the line with highest YdPl among common bean parental lines in GH1. For GH2, SEN118 was the best common bean parental and presented an YdPl of 9.46 g/plant. In GH2, 19 interspecific lines outperformed SEN118 and 125 interspecific lines were superior to SEF10 (the second-best common bean parental with yield of 9.46 g/plant). Among the 107 lines from GH1 and 125 lines from GH2, 66 lines were found in both outperforming groups.

### Genotyping and population structure

The IMAWT population was genotyped by GBS and after filtering low quality, highly heterozygous and rare variants obtained a total of 24.205 bi-allelic SNPs for 236 interspecific lines and the eight parental lines mentioned previously. The number of SNPs per chromosome ranged between 877 and 6,720 SNPs (chromosome 09 and 03, respectively). The SNPs’ distribution inside each chromosome was heterogeneous with higher marker density in the telomeric regions (**Supplementary Figure 6**).

**Figure 4:**
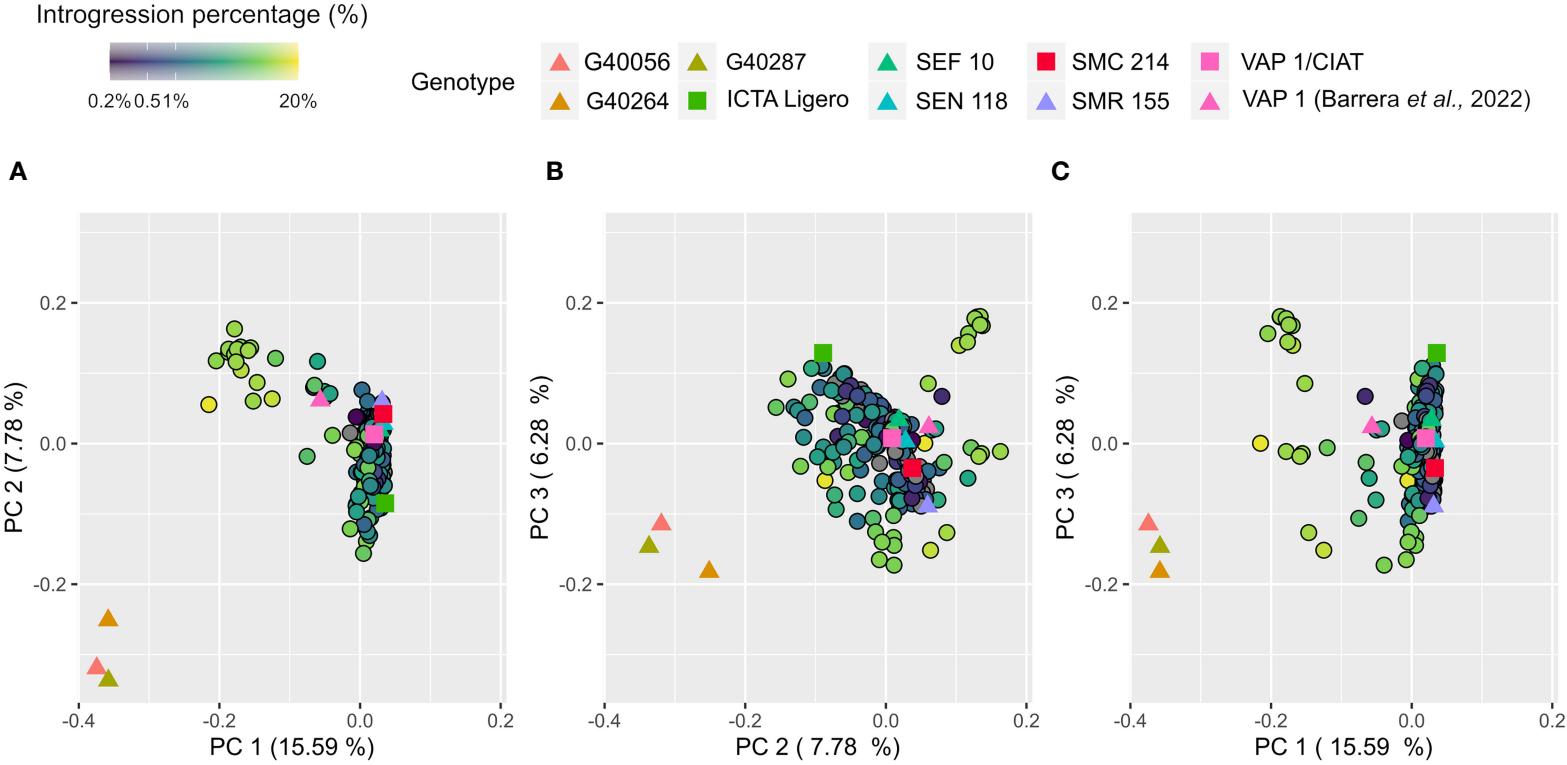
Principal component analysis of 263 samples using 24.205 biallelic SNPs. Markers were colored according to the introgression percentage obtained from introgression analysis. Parental lines were highlighted with different colors.

Population structure was assessed through Principal Component Analysis (PCA). The first three principal components (PC) explained 29.7% of the total variance (**Supplementary Figure 7**). The first two PCs differentiate the wild relatives’ samples (G40056, G40287 and G40264) from the common bean primary genepool. In between both groups a group of interspecific lines was located resembling similarities from both (**Figures 4A, B**). The PC3 allowed the differentiation inside the *P. vulgaris* primary genepool observing contrasting genetic differences between ICTA Ligero and SMR155 (**Figure 4C**).

### Introgression analysis

**Figure 5:**
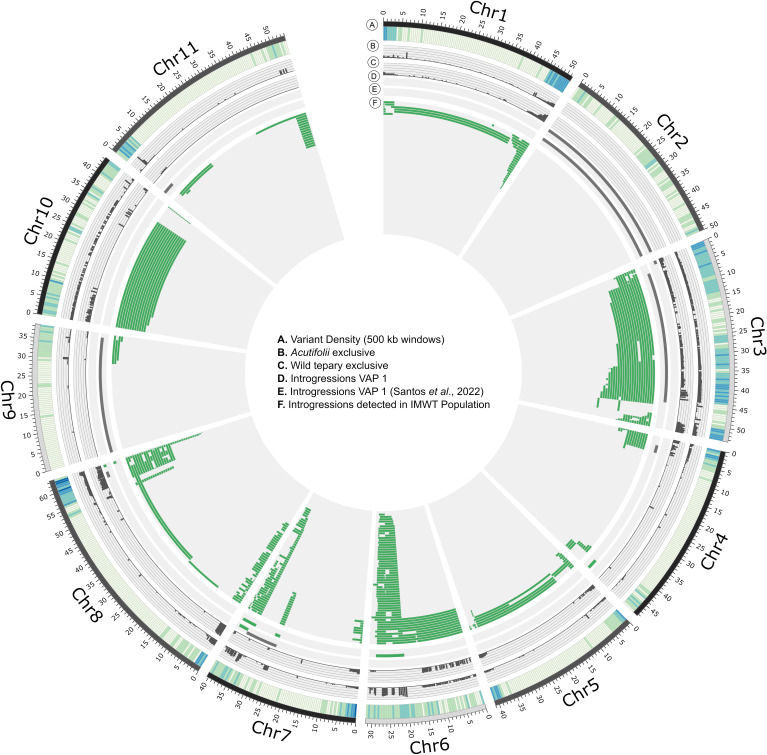
Genome-wide distribution of *Acutifolii* interspecific introgressions in VAP 1 and IMAWT population. The rings of the circle show **(A)** variant density of full genotype matrix, **(B)** Absolute frequency of samples carrying *Acutifolii* alleles of selected subset, **(C)** Absolute frequency of samples carrying wild *P. acutifolius alleles* of selected subset, **(D)** detected introgressions for VAP 1 sequenced in this study and **(E)** of sample provided by Barrera et al. (2022). **(F)** *Acutifolii* introgressions detected in IMAWT Population. Green tiles represent homozygous *Acutifolii* introgressions and grey tiles undefined regions.

The IMAWT population presented interspecific introgressions from wild tepary lines (G40056 - G40287) and *P. parvifolius (*G40264) from the bridging genotype VAP1. A subset of contrasting 7,915 bi-allelic SNPs were selected to detect the introgressions (**Supplementary Figure 3**). Those contrasting variants provide evidence of the location and extent of introgressions present in the population. For each variant we counted the number of samples that carried at least one *Acutifolii* allele (**Figure 5B**). The selected SNPs’ distribution was uneven along the genome. We observed in chromosomes 02, 09, and 11 a low density (17, 123, and 78 SNPs/chromosome, respectively) whereas chromosomes 01, 03, and 08 presented higher densities (1.219, 2.807 and 1.106 SNPs/chromosome, respectively; **Supplementary Table 4**). The crossing-over points between *Acutifolii* and *P. vulgaris* backgrounds were detected in each sample resulting in 465 homozygous introgression events in 203 interspecific lines. Additionally, a total of 309 regions was detected that were due to high heterozygosis, or background changes below the minimum introgression size were labeled as undefined (**Supplementary Figure 8**). For those families carrying at least one introgression event, the introgression percentage by sample (IP = Total introgression length/genome size) varied between 0.03 and 17.13%. Half of those families presented an IP below 1.8%, and GCDT 237 showed the highest IP (**Supplementary Figure 9**). Chromosomes 01, 03, 05, 06, 08, and 10 were covered almost completely by introgressions (86.7% - 100% of coverage). Chromosomes 04, 07, and 09 presented a low introgression coverage (25% - 26.5%). No introgression events were detected in chromosome 02 (**Figure 5**). With the IMAWT population it was possible to cover 59.8% of the common bean with wild introgressions (**Supplementary Table 4**).

For the IMAWT population we detected introgressions in 54 and 46 samples for chromosomes 06 and 07 (20 – 29 Mb and 36 − 39 Mb, respectively), and importantly VAP1 presented introgressions events in the same regions indicating the putative origin (**Figures 5D, E**). We selected a subset of 1,860 variants where wild tepary accessions didn’t share any alleles with the *P. vulgaris* parental lines, G40264 and VAP1 samples to confirm if the population carries wild tepary-specific alleles (**Supplementary Figure 3**). We counted the number of introgression lines that carried at least one wild tepary allele and found they were scattered throughout the genome, excepting chromosome 02 (**Figure 5C**).

**Figure 6:**
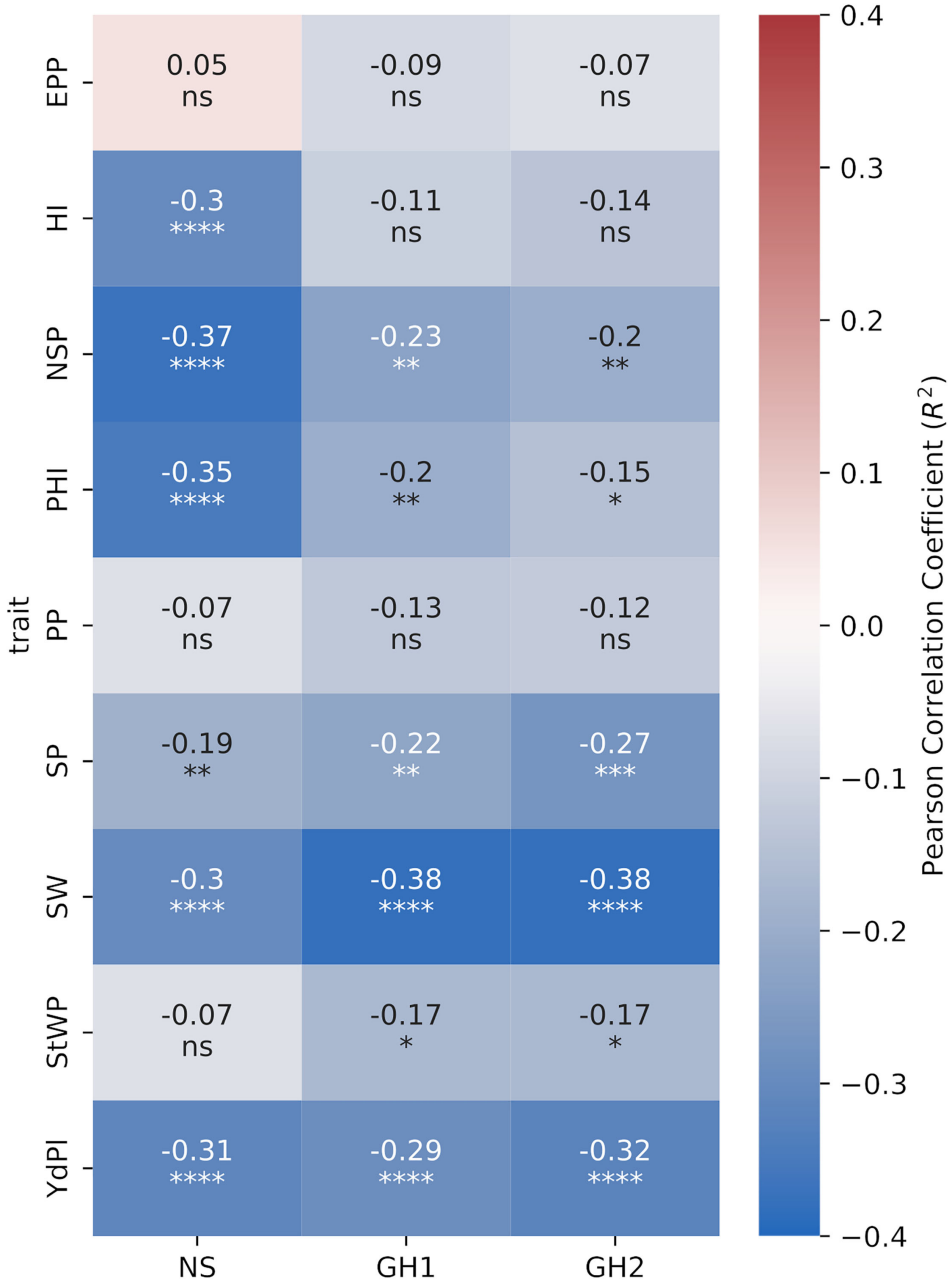
Pearson correlation heatmap of introgression percentage by sample (IP) carried at least one introgression event with traits for the subset of 185 samples. EPP: Empty pods per plant. NSP: Number of seeds per pod. PHI: Pod harvest index. PP: Pods per plant. SP: Seeds per plant. SW: Seed weight. StWP: Dry stem weight per plant. YdPl: Yield per plant. Asterisks indicate the significance of Pearson correlation coefficients ns P > 0.05; ^*^P ≤ 0.05; ^**^P ≤ 0.01; ^***^P ≤ 0.001; ^****^P ≤ 0.0001.

We selected 203 interspecific lines where introgression analysis detected at least one introgression event and correlated introgression percentage (IP) with measured quantitative traits for each environment. In the NS environment the correlations were higher (more negative and significant) for NSP, PHI and HI than in both HS environments where were not significant for HI. In contrast, correlations of IP to PP, SP, SW, and StWP were lower in NS than in both HS environments. For YdPl the correlations were similar across all environments (**Figure 6**).

### Genome-wide association analysis

Genetic association analyses were performed using the FarmCPU method, and significant associations or Quantitative Trait Nucleotides (QTNs) were selected according to the Bonferroni threshold (-log_10_(P-value) > 5.7). In total, 27 QTNs were detected for five traits (EPP, YdPl, StWP, NSP, and SW). In HS environments, we detected 24 QTNs (14 and 10 for GH1 and GH2) and 3 in NS (**Supplementary Figure 10**).

EPP showed the highest number of significant associations detecting 9 QTNs (5 and 4 for GH1 and GH2, respectively; **Supplementary Figures 10, 11**). EPP1.1 and EPP1.2 were detected independently in GH1 and GH2, respectively, and were separated by 29 bp. The minor allele for both QTNs was inherited from wild tepary parental lines presenting a positive allelic effect of 0.67 and 1.02 empty pods/plant and explaining 10.4 and 14.5% of the phenotypic variance, respectively (**Table 1**). All the QTNs presented a positive allelic effect except EPP6.4 (-1.76 empty pods/plant), in which the minor allele was inherited from ICTA Ligero.

**Table 1:** Detected QTNs above Bonferroni significance threshold of GCDT population evaluated in three different environments.

SNP	Name	MAF	Effect	Location	-Log_10_(P)	PVE	G40056	G40287	ICTA Ligero	SEF 10	SEN 118	SMC 214	VAP 1	SMR 155
Pv2.1_Chr01_1626829_A/G	EPP1.1	3%	0.67	GH1	6.25	10.4	G/G	G/G	A/A	A/A	A/A	A/A	A/A	A/A
Pv2.1_Chr01_1626868_C/T	EPP1.2	3%	1.02	GH2	8.35	14.5	T/T	T/T	C/C	C/C	C/C	C/C	C/C	C/C
Pv2.1_Chr01_5816071_G/C	EPP1.3	49%	0.34	GH2	6.5	0.3	C/C	C/C	G/G	C/C	C/C	G/C	G/C	G/G
Pv2.1_Chr01_50793604_T/C	StWP1.1	9%	0.57	GH1	7.03	2.1	./.	C/C	T/T	T/T	T/T	T/T	T/T	T/T
Pv2.1_Chr02_38513296_A/G	SW2.1	18%	-0.79	NS	5.86	2.4	./.	./.	A/A	A/A	A/A	A/A	G/G	A/A
Pv2.1_Chr02_49445611_C/T	YdPl2.1	5%	1.43	GH1	8.07	10.4	T/T	T/T	C/C	C/C	C/T	C/T	C/C	C/C
Pv2.1_Chr03_28779364_T/C	YdPl3.2	13%	1.61	NS	7.72	15.7	C/C	C/C	T/T	T/T	T/T	T/T	T/T	T/T
Pv2.1_Chr03_36852741_C/A	StWP3.2	5%	-0.77	GH2	6.96	5.2	C/C	./.	C/C	C/C	C/C	C/C	C/C	C/C
Pv2.1_Chr03_38826360_T/C	StWP3.3	7%	-0.6	GH1	5.94	5.4	T/T	T/T	T/T	T/T	T/T	T/T	T/T	T/T
Pv2.1_Chr03_41761731_A/G	NSP3.1	10%	0.32	GH1	6.31	21.8	A/A	A/A	A/A	A/A	A/A	A/A	A/A	A/A
Pv2.1_Chr03_52066941_G/A	StWP3.4	7%	0.86	GH2	10.87	7.1	./.	A/A	G/G	G/G	G/G	G/G	G/G	G/G
Pv2.1_Chr04_47499464_C/T	NSP4.2	18%	0.21	GH1	5.73	6.1	C/C	C/C	C/C	T/T	./.	T/T	T/T	T/T
Pv2.1_Chr06_14973060_T/C	StWP6.5	28%	-0.49	GH2	7.03	6.7	T/T	T/T	T/T	T/T	T/C	T/T	./.	C/C
Pv2.1_Chr06_19766042_C/T	EPP6.4	11%	-1.76	GH1	7.1	17.2	C/C	C/C	T/T	C/C	C/C	C/C	C/C	C/C
Pv2.1_Chr06_19766268_A/C	EPP6.5	11%	0.31	GH1	8.88	19.4	C/C	C/C	A/A	C/C	C/C	C/C	C/C	C/C
Pv2.1_Chr06_23874717_C/T	YdPl6.3	49%	-0.65	NS	6.72	5.9	T/T	T/T	C/C	T/T	C/C	C/C	./.	C/C
Pv2.1_Chr07_2538704_A/G	SW7.2	3%	-2.2	NS	7.7	46	A/A	G/G	A/A	A/A	A/A	A/A	A/A	A/A
Pv2.1_Chr07_33348492_A/T	EPP7.6	31%	0.36	GH2	5.8	1.5	A/A	A/A	A/A	A/A	T/T	./.	T/T	A/A
Pv2.1_Chr07_37393376_C/G	YdPl7.4	15%	0.74	GH2	6	4.7	G/G	G/G	C/C	C/C	C/G	C/C	C/C	C/C
Pv2.1_Chr07_37574404_G/A	EPP7.7	19%	0.43	GH1	10.36	2.5	G/G	G/G	A/A	A/A	A/A	A/A	./.	A/A
Pv2.1_Chr08_334241_T/C	StWP8.6	10%	0.51	GH1	6.94	6.9	./.	C/C	T/T	T/T	T/T	T/T	T/T	T/T
Pv2.1_Chr08_3658229_A/T	YdPl8.5	2%	2.25	GH1	7.08	23.3	A/A	A/A	T/T	T/T	T/T	T/T	T/T	T/T
Pv2.1_Chr08_3658229_A/T	YdPl8.5	2%	2.07	GH2	6.62	46	A/A	A/A	T/T	T/T	T/T	T/T	T/T	T/T
Pv2.1_Chr08_50339512_C/T	YdPl8.6	3%	2.25	GH1	9.94	14.8	C/C	C/C	T/T	T/T	T/T	T/T	T/T	T/T
Pv2.1_Chr08_60092300_C/A	EPP8.8	3%	0.85	GH2	7.35	26.7	C/C	C/C	C/C	C/C	C/C	C/A	C/C	C/C
Pv2.1_Chr09_36881844_C/T	StWP9.7	3%	1.5	GH2	8.69	16.2	T/T	T/T	T/T	T/T	T/T	T/T	C/T	T/T
Pv2.1_Chr10_6700431_C/T	YdPl10.7	3%	-1.07	GH1	5.92	7.1	C/C	C/C	C/C	C/C	C/T	C/T	C/C	C/C
Pv2.1_Chr11_6795744_A/G	EPP11.9	12%	0.39	GH1	6.29	2.3	G/G	G/G	A/A	A/A	A/A	A/G	A/A	A/A

**SNP:** QTN id indicating the reference genome, chromosome number, reference and alternative allele. **MAF,** Minor allele frequency (%); **Effect,** Allelic effect relative to minor allele; **LogP,** -log_10_(p-value); **PHI**, Pod Harvest Index (%); **YdPl**, Yield per plant (g/plant); **EPP**, Empty pods per plot (pods/plot); **PN,** Pod number (pods/plot); **NSP,** Average number of seeds per pod; **SW,** Seed weight (g/100 seeds)**; PVE**, Percentage of phenotypic variance explained (%);^3^Estimated allelic effect relative to minor allele. Genotype call for each parental line were indicated. Green cells represent the minor allele (less frequent allele among the population).

We detected 8 QTNs for YdPl (2 in NS, 4 in GH1, and 2 in GH2) and the most significant association was observed for QTN YdPl8.6 (-log_10_(P-value) = 9.94). Jointly with YdPl3.2 and YdPl8.5 (which was detected independently in both HS environments) in these three QTNs the minor allele was inherited from wild tepary parental lines and the estimated allelic effects ranged between 2.07 and 2.25 g/plant. YdPl6.3 and 10.7 presented a negative allelic effect reducing the YdPl by -0.65 and -1.07 g/plant (**Table 1**). Notably, QTNs near to YdPl8.6 existed at not significant levels but still highly associated with StWP (at 628 kb of distance) in GH1 and in the same position in GH2 (**Supplementary Figures 10, 12**).

For StWP, 7 QTNs were detected (3 and 4 in GH1 and GH2, respectively). The most significant association was observed for QTN StWP3.4. Jointly with StWP1.1, and StWP8.6, these are the QTNs whose minor allele was inherited from tepary parental lines, and the estimated allelic effect is positive, ranging between 0.57 to 0.86 g/plant (**Table 1** **;** **Supplementary Figures 10, 13**). StWP3.2 and StWP3.3 were monomorphic within parental lines. The origin of these putative alleles could be traced back to a duplicate of VAP1, indicating heterozygosity of the bridging genotype when crosses were performed (**Supplementary Figures 14, 15**)

Two QTNs NSP3.1 and NPS4.2 were detected in GH1, and the estimated allelic effect was 0.32 and 2.1 seeds/pod, respectively (**Table 1**; **Supplementary Figures 10, 16**). NSP3.1 was monomorphic within parental lines. Similarly, to StWP3.2 and StWP3.3, the minor allele was observed in a duplicate of VAP1 (**Supplementary Figure 17**). The minor allele of NSP4.2 was present in both tepary parental lines and ICTA Ligero (**Table 1**).

SW2.1 and SW7.2 were detected in the NS environment. The estimated allelic effect was negative in both cases, ranging between -0.79 to -2.20 g/100 seeds (**Table 1** **;** **Supplementary Figures 10, 17**). For SW2.1, the minor allele could be inherited from VAP1 or also possibly from wild tepary parental lines. For SW7.2, the minor allele was inherited from G40287 (**Table 1**).

## Discussion

Climate change threatens current and future food security, including in regions using common bean as a staple crop. Predictions for common bean growing areas of southeastern Africa state more frequent scenarios of elevated temperatures (heat) and reduced rainfall (drought) that will become unsuitable for bean cultivation by the year 2050 (Hummel et al., 2018). Crop wild relatives and landraces historically adapted to arid or semi-arid conditions are promising sources of useful variation (Cortés & López, 2021). Tepary bean (*P. acutifolius*) is a wild relative of common bean adapted to desert and semiarid environments (Freeman, 1912). The use of tepary bean for common bean breeding was first reported by Honma (1956) who was looking for resistance to common bacterial blight (CBB; caused by *Xanthomonas campestris*). Honma developed an interspecific population using embryo rescue and used recurrent backcrossing with the common bean (Honma, 1956). More recently interspecific populations between tepary and common bean with useful variation for cold, drought and bruchid resistance have been reported (Martinez, 2010; Souter et al., 2017).

The population structure of IMAWT locates the wild tepary bean accessions close to G40264 (*P. parvifolius*) and more distantly to common bean parental lines. This is in accordance with the current taxonomy of the genus *Phaseolus*, which places these two crop wild relatives in the section named *Acutifolii* distanced from *Phaseolii* section to which *P. vulgaris* belongs (Freytag & Debouck, 2002). We located a group of ILs resembling both genetic backgrounds between these two clusters, indicating the presence of introgression fragments from the *Acutifolii* wild relatives. To detect the introgressions, we selected a subset of 7,915 SNPs that were contrasting between common bean parental lines and *Acutifolii.* This approach has been previously implemented for interspecific biparental populations in maize (*Zea mays*), melon (*Cucumis melo*) and tomato (*Solanum lycopersicum* L.) (Gonda et al., 2019; Kolkman et al., 2020; Oren et al., 2020).

We detected 465 homozygous introgressions events carried by 203 ILs that jointly covers with at least one introgression the 59.8% of the common bean reference genome. We observed that for centromeric regions there is a lower frequency of introgressions, probably due to the natural low recombination rate of centromeric regions (Schmutz et al., 2014). The lack of introgressions in chromosome 02 and 09 is in line with the reproductive isolation QTLs reported by Soltani et al. (2020). In a BCF_1_ biparental population derived from ICA Pijao x Fijol Bayo (domesticated tepary bean), they observed an absence of recombination in the first 33 Mb of chromosome 02 and first 22 Mb of chromosome 09. They thus concluded that tepary bean carries chromosomal rearrangements in those regions that presumably cause hybrid sterility (Soltani et al., 2020). Notably, ICA Pijao is a genotype widely recognized for its reproductive compatibility with tepary bean, producing vigorous hybrid plants after embryo rescue (Parker & Michaels, 1986). This latter genotype is present in the interspecific line INB834 used twice in the VAP1 pedigree suggesting that G40264 (*P. parvifolius*) not only contributes to reproductive compatibility but also ICA Pijao.

We observed that IMAWT population presented a higher introgression detection rate in the regions predicted as introgression for VAP1 samples, indicating that the latter genotype is also a source of introgressions. The distal arm of both chromosomes 06 and 07 presented a high frequency of introgressions among the population, but no wild tepary exclusive variants were located, suggesting that those introgressions might be provided by G40264 through VAP1. In contrast, the distal end of chromosome 08 VAP1 presented an introgression where multiple exclusive wild tepary variants were located, suggesting that VAP1 also carries introgressions from the tepary bean. The origin of this introgression could be traced back to INB841, which is a VAP1 parent, where a tepary introgression has been reported in the same location (Lobaton et al., 2018). There were also other regions where VAP1 introgression was detected, and multiple ILs were observed carrying wild tepary bean exclusive variants’ alleles indicating that recombination had occurred between wild tepary accessions and VAP1, as in chromosome 01, 05, 10 or the distal end of chromosome 03.

### Physiological response of IMAWT population

Our results indicate that the population developed under heat stress conditions displayed a reduction of YdPl and NSP between 5-6% and 9-14%, respectively. Similarly, in HS vs NS environments we observed an increase of StWP and EPP which indicated by a prolific branching and production of empty and malformed pods (EPP). Similar observations have reported an intense vegetative growth in common beans under high night temperatures of 27°C. These indicated that yield was constrained by an increase in flower bud abscission, in flowers, and in young pods rather than a reduction in flower production itself (Konsens et al., 1991).

Yield reductions estimated in this study are lower compared to previous reports. In field conditions Vargas et al. (2021) evaluated an Andean population in multiple locations in Colombia, comparing hot (34/23°C; average temperature day/night) vs control (30/19°C) environments, observing average yield reductions between 26 and 37% (Vargas et al., 2021). A study under greenhouse conditions reported even more severe heat stress effects with yield reductions between 77 and 98% for NS (27/21.1-22.9°C) vs HS (29/22.9°C) (Porch, 2006). The differences in yield reductions could be attributed to multiple environmental factors, including the reduction of the incident sunlight, in this case being 47% lower in an HS than in an NS environment, and logically also the temperature.

We observed a negative correlation between IP and yield components for all environments, indicating a negative effect of those introgressions over the plant performance. In NS, the correlations were higher in a magnitude than HS environments particularly for PHI, HI, NSP and YdPl. The observed negative effects in NS could arise from conflicts between both distant genomes but also due to the tepary natural adaptation to hot and dry environments (Bitocchi et al., 2017). Specialized genes for heat or drought resistance could be inherited but the same genes could have a trade-off effect in non-heat or in humid contexts. In each greenhouse we observed interspecific lines that outperformed the best common bean parental lines in yield indicating the existence of useful variance for heat resistance.

### GWAS analysis

GWAS analyses in common bean using Andean and Mesoamerican germplasm collections offered a broad perspective on common bean diversity and the genetic basis of multiple traits such as growth habit, seed size, and drought tolerance (Hoyos-Villegas et al., 2017; Diaz et al., 2020; Valdisser et al., 2020). We used the method FarmCPU to perform the GWAS analyses, this method uses a fixed effect model (FEM) to test the association one marker at time using as covariates a multiple associated marker set (also named pseudo QTNs) to control false positives. To avoid overfitting pseudo QTNs are optimized by a random effect model (REM) based on the testing statistics (P-values) and positions by using the SUPER algorithm a bin-based algorithm used for selecting across the whole genome multiple associated QTNs (X. Wang et al., 2014; Liu et al., 2016). Wild tepary parental lines presented wide genetic and phenotypic differences in relation to the remaining parental lines and the population itself. Besides the ability to control the false positive of FarmCPU we decided to remove tepary parental lines of the GWAS analyses due to an inflated trend in P-value QQ-plot when are included (data not shown).

Testing association one marker at time results in a multiple testing problem causing spurious associations. To overcome this, we used the Bonferroni threshold to differentiate the true positives from false positives, this threshold is considered the most conservative method and depends on genotype density and the desired significance level (Kaler & Purcell, 2019). Genotype density reported in other studies that used GBS and the RE ApeK1 produced between 20 – 30k SNPs (Diaz et al., 2020; García-Fernández et al., 2021). Exist other GBS protocols that use different RE enzymes or dual digest with MseI and TaqαI that could increase in 3.8 to 12.5-fold the number of SNPs compared to ApeK1 (Schröder et al., 2016).

High rates of missing data are a major thread for GWAS statistical power or ability to identify true positives (Korte & Ashley, 2013). Is common the presence of missing SNP calls in GBS datasets due to presence-absence variation of cutting site, differential methylation, lower library quality and sequence coverage (Poland & Rife, 2012; Mohammadi et al., 2019). Commonly this is resolved *via* imputation of missing data but error rate of imputation increase as the MAF decrease (Marchini & Howie, 2010). We selected a low MAF threshold (2.5%) to avoid remove introgression sites because we expected a low frequency due to the crossing scheme that included two crossing cycles with common bean diluting the wild tepary contribution in the offspring.

With the GWAS analyses we reported 27 QTNs. From these QTNs, the less frequent allele could be traced back to the tepary parental lines in nine cases indicating that were located inside a tepary introgression segment. Three presented a detrimental effect in terms of increasing empty pod numbers (EPP1.1 and EPP1.2) or decreasing seed weight (SW7.2) and a favorable effect increasing yield (YdPl3.2, YdPl8.5 and YdPl8.6) and stem production (StWP1.1, StWP3.4 and StWP8.6).

In the proximal arm of chromosome 01 were detected two QTNs for EPP inside tepary introgressions (EPP1.1 and 1.2). A QTL covering the latter QTNs named PdShr1.1 located between the positions 1.4 - 1.65 Mb for shriveled seeds in common bean under high temperatures had been reported (Vargas et al., 2021). EPP1.1 and 1.2 located in the gene model Phvul.001G020000 annotated as a GPI inositol-deacylase or PGAP1-like protein which is related with the endoplasmic reticulum export of proteins to the cell surface (Mañuel & Howard, 2016). For QTN EPP7.7 with exception of VAP1 all the parental lines were genotyped and observing in homozygous state the alternative allele in tepary lines. This QTN located in the gene model Phvul.007G254000 which presents a S-locus glycoprotein domain (PF00954) involved in pollen recognition system to avoid self-fertilization in the Brassicaceae family (Hinata et al., 1995).

The seed weight of wild *P. acutifolius* parental lines is significantly lower in comparison to domesticated common bean parental lines. Here we report SW7.2 a tepary introgression QTN that significantly reduce the seed size in NS environment. SW7.2 is in the gene model Phvul.007G031800 annotated as alpha-trehalose-6-phosphate synthase (TPS) which catalyzes the synthesis of alpha-trehalose-6-phosphate (T6P) a signaling molecule that significantly affects the regulation of carbon allocation and utilization in plants (Paul et al., 2018). Valdisser et al. (2020) reported a common bean QTN for seed weight detected under drought and irrigated conditions at 1 Mb away of SW7.2.

Three QTNs with positive effect for YdPl in tepary introgressions were reported in this study. YdPl3.2 was detected in NS environment, inside the gene model Phvul.003G112400 annotated as ATP synthase gamma-related. In a QTL analysis for fatty acids of seed in soybean (*Glycine max*) two QTLs for palmitic and oleic acid content had been reported and in a centered window of 1 Mb on those QTLs was present the gene Glyma.17G032400 which is ortholog to Phvul.003G112400 (Smallwood, 2015). In a GWAS study with a Chinese diversity panel of common bean were reported at 460 kb downstream and 717 kb upstream of YdPl3.2 QTNs for days to flowering, growth habit and plant height (Wu et al., 2020). The QTNs YdPl8.5 and 8.6 were detected in HS environments inside the gene models Phvul.008G042800 and Phvul.008G177800 annotated as polyvinyl-alcohol oxidase and E3 ubiquitin-protein ligase, respectively. Protein ubiquitination is the major eukaryotic proteolytic pathway responsible of degradation of misfolded proteins (Zhang et al., 2021). Multiple reports states that members E3 ubiquitin ligases enhance the thermal resistance in plants regulating the activity of calcium channels, transcription of heat shock proteins and closure of stomata (Z. Bin Liu et al., 2014; J. Liu et al., 2016). Is important to mention that at 7 kb upstream of YdPl8.5 is located the gene model Phvul.008G043000 which present a WRKY DNA-binding domain present in transcription factors involved in various developmental and physiological process but particularly prominent in the modulation of response to biotic and abiotic stress (Jiang et al., 2017; Cheng et al., 2021). The QTN YdPl7.4 was detected with the phenotype data of GH2 environment at the distal end of chromosome 07. Tepary parental lines were homozygous for the alternative allele but also SEN118. This QTN located in the gene model Phvul.007G251800 a NADPH-dependent thioredoxin reductase 3 (NTRC) which helps to maintain a reduced cellular environment and protect against oxidative stress during stress events and had been reported to be overexpressed in heat stress in common bean (Soltani et al., 2019).

Three QTNs with positive effect inside tepary introgressions were detected for StWP. StWP3.4 is located inside the gene Phvul.003G282900 that belongs to RmlC-like cupins superfamily proteins. Is important to mention that this gene presented a differential expression between a terminal drought stress and non-stress environments in common bean (Subramani et al., 2023). StWP8.6 is located in the gene model Phvul.008G003600 and is flanked downstream by the gene Phvul.008G003500 nitrate transporter protein-peptide transporters (NTR1-PTR) and upstream by the genes Phvul.008G003700 and Phvul.008G003800 two flowering locus T like proteins (FT-like). The FT-like genes are identified as a major regulatory factor in a wide range of developmental process including fruit set, vegetative growth, stomatal control and tuberization (Pin & Nilsson, 2012). By the other hand the Chiba et al. (2015) reports that NRT1(PTR) plays an important role transporting phytohormones even during the stress conditions such as auxins, abscisic acid and gibberellins. A QTL named DPM8.1 that covers the latter gene model is related with days to physiological maturity in common bean (Diaz et al., 2020).

### Future perspectives

The detected QTNs have the potential to broaden the genetic base in domesticated common bean genepools and support the strategy of incorporating functional genetic variation to increase heat tolerance from wild crop relatives (Tanksley & McCouch, 1997). Thus, it is necessary to further confirm if the introgression segments that include those QTNs can in fact host genes involved in adaptation to heat stress. Is important to validate the input from single introgression fragments, by revealing their effect in homogeneous genetic backgrounds, avoiding the possible interaction with other introgression events that diminish yield. An alternative approach would be testing contrasting lines with and without the QTNs under natural conditions. Therefore, further filtering and characterization of the genome regions exclusively related to heat stress should be identified. Applying cutting-edge techniques like long read sequencing can improve our understanding of interspecific introgression and improve breeding effectiveness. Long read sequencing will facilitate whole genome assemblies of parental and bridge lines, revealing hidden genomic regions masked by the reference genome utilized. Discovering the exact crossing over points, with higher resolution to identify smaller introgressed regions that cannot be found by SNPs matrixes will help validate wild introgression inputs. Regardless, this genome-wide association study on interspecific common bean populations derived from wild *Phaseolus* relatives has revealed improved heat resistance as a result of successful genetic introgression between two *Phaseolus* sister species, demonstrating better performance under heat stress conditions. The allele diversity from wild materials increases the adaptability of domesticated plants *via* enhanced biotic and abiotic stress resistance, especially in the context of climate change and potential pathogen outbreaks. The results from this study broaden our understanding of genetic crossing using bridging interspecific lines applied to heat-tolerant populations through recognizing the wild introgression segment using GBS sequencing on introgression lines.

These results represent an important contribution to common bean genetic improvement. In the short term, QTL for heat tolerance may advance on-going work for improved bean crop adaptation in lowland environments in Central America, the Caribbean, and East, southern and West Africa. In the medium to long term, characterizing introgression from sister species *P. acutifolius* and *P. parvifolius* can open new perspectives for managing a range of abiotic and biotic factors that limit bean productivity and production. These sister species evolved in hot, dry environments which may become more prevalent in the future in bean production regions, and for adaptation to which genetic diversity in *P. vulgaris* is limited. This study’s findings will facilitate broadening this introgression.

## Nomenclature

BLUPs, Best Linear Unbiased Predictors; CIAT, International Center for Tropical Agriculture; CTAB, Ionic detergent cetyltrimethylammonium bromide; DAS, Days After Sowing; EPP, Empty Pods per Plot; EU, Experimental Unit; GBS, Genotyping by sequence; GH1, Greenhouse 1; GH2, Greenhouse 2; H2, Heritability in broad sense; HI, Harvest Index (%); HS, Heat stress environments (GH1, GH2); ILs, Introgression Lines; IMAWT population, Interspecific Mesoamerican Wild Tepary population; IP, Introgressed Percentage (%); QTN, Quantitative Trait Nucleotide; MAF, Minor Allele Frequency (%); MLM, Mixed Linear Model; NS, Non heat stress environment; NSP, Number of seeds per pod; PAR, Photosynthetic Active Radiation; PCA, Principal Components Analysis; PHI, Pod Harvest Index (%); PP, Pods per plant; QTL, Quantitative trait loci/locus; QTNs, Quantitative trait nucleotides; SNPs, Single Nucleotide Polymorphism; SP, Seeds per plant; STI, Stress Tolerance Index; StWP, Dry weight of leafless stems per plot; SW, Seed Weight (g/100 seeds); VAP, Vulgaris-Acutifolius-Parvifolius bridging genotype YdPl, Yield per plant (g/plant)

## Data availability statement

The datasets presented in this study can be found in online repositories. The names of the repository/repositories and accession number(s) can be found below: https://www.ncbi.nlm.nih.gov/, PRJNA873092.

## Author contributions

SC (selection of population, phenotypic sampling, DNA library preparation, bioinformatic analysis and article writing). MU (phenotyping experiment coordinator, editing, and reviewing the article), DA-S (support in statistics and bioinformatics, editing, and reviewing the article), BR (research coordinator, population selection, reviewing the article), JL (research coordinator and article writing), JA (support in statistics), GM (editing and reviewing the article, and providing funds). SB (supported the original development of mapping population, research coordination, editing, and reviewing the article). All authors contributed to the article and approved the submitted version.
