# Supplementary material for: Interspecific common bean population derived from Phaseolus acutifolius using a bridging genotype demonstrate useful adaptation to heat tolerance
Source: Front Plant Sci. 2023 May 12;14:1145858. doi: 10.3389/fpls.2023.1145858 (PMC10246688; doi:10.3389/fpls.2023.1145858)
Supplement: Supplementary file 1 [file DataSheet_1.zip › Image 1.pdf]

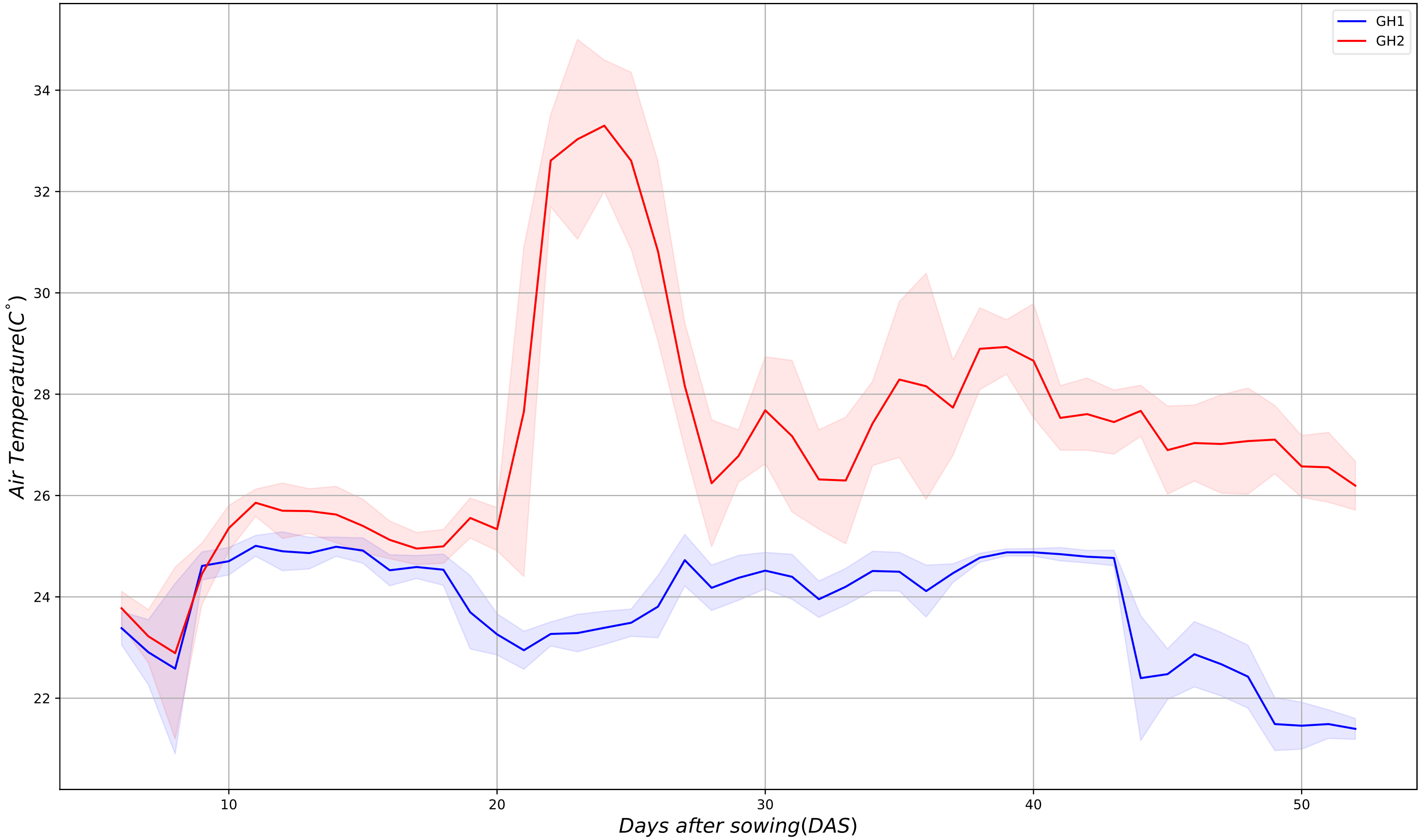

Supplementary Figure 1: Comparison of night time temperatures in the greenhouses where population was screened in $F_{4:5}$. *The daily average of temperatures between 18:00 and 06:00 was calculated. The shading around the lines corresponds to the standard deviation

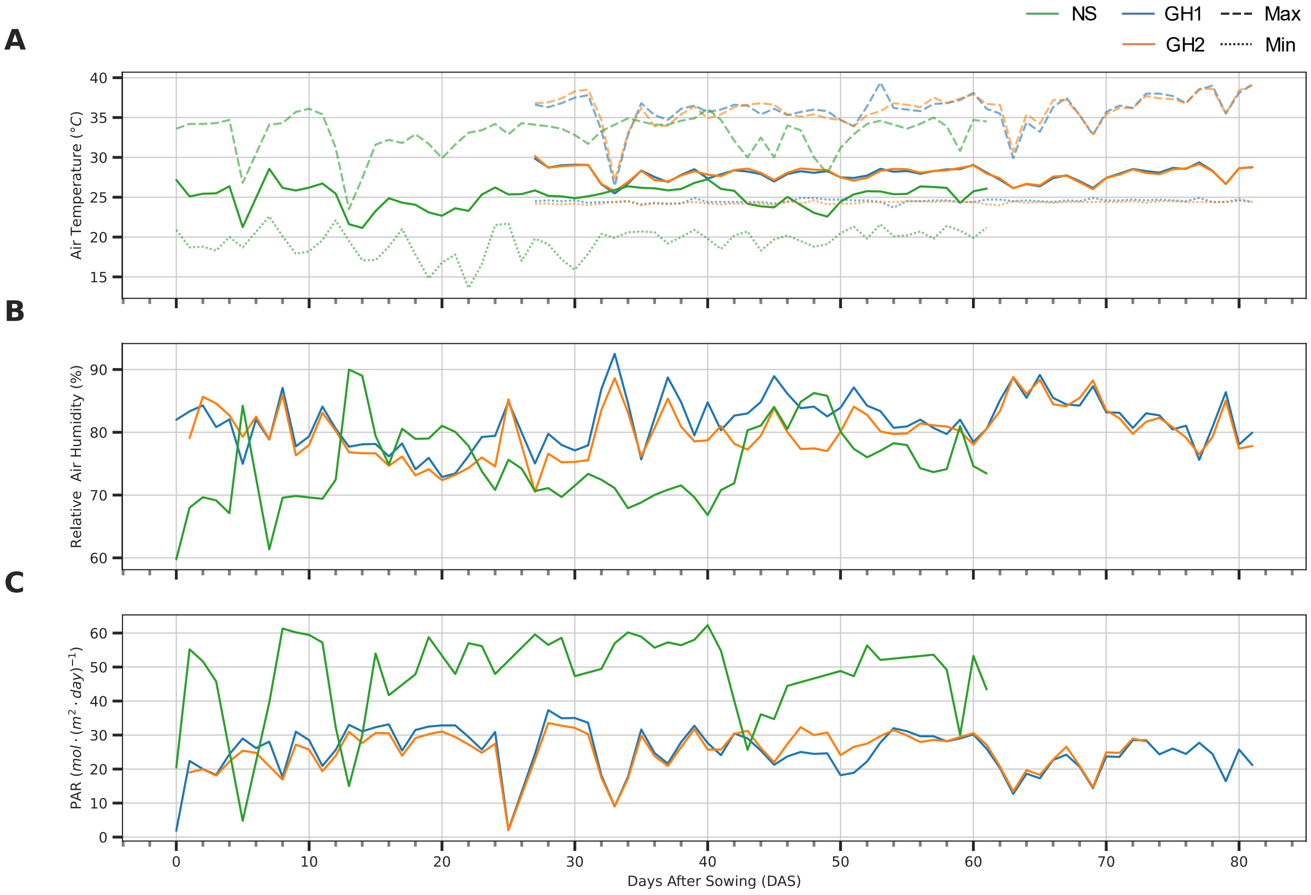

Supplementary Figure 2: Climatic parameters by day measured over the cultivation period connected to the data in the manuscript (F5:6 generation). A: Air temperature (°C). B: Relative air humidity (%). C: Photosynthetic Active Radiation (PAR) accumulated by day (mmol/m^2*day). Color of line represents the environment. Green for NS, blue for GH1 and orange for GH2. Trace of line indicate a descriptive parameter. Solid line average of day, dotted and dashed line minimum and maximum value measured in the day.

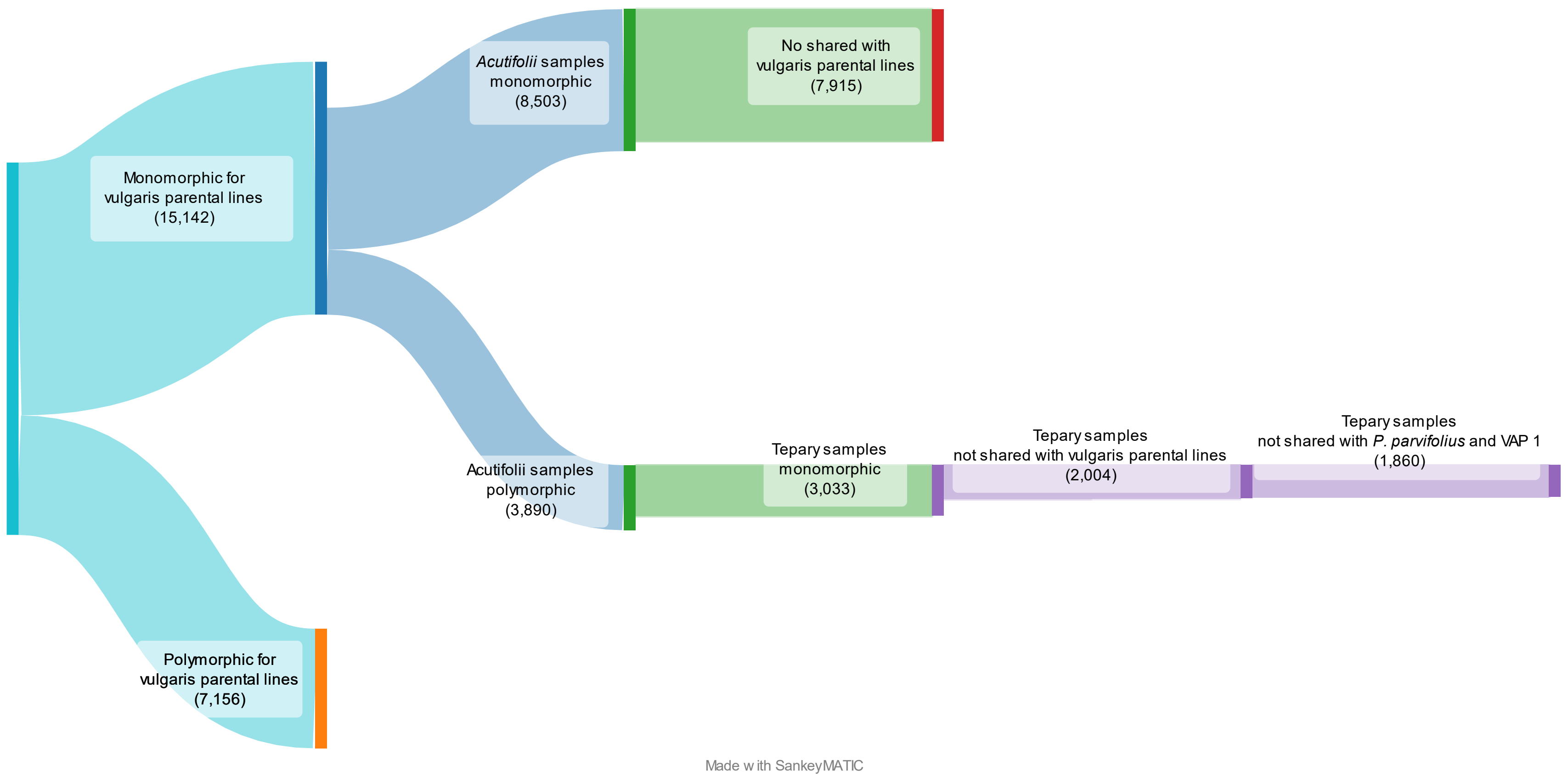

Supplementary Figure 3: Sankey diagram showing the selection process of contrasting SNPs for introgression analysis. Original dataset is composed by 24.205 *bi-allelic SNPs*. Were selected the variants where the five common bean parental lines (ICTA Ligero, SEF10, SMR155, SMC214 and SEN118) present no missing data and monomorphic. From those were selected the variants where *Acutifolii* samples (*G40056, G40287 and G40264*) present no missing data, homozygous and don't share any allele with common bean parental samples (8.503 SNPs).

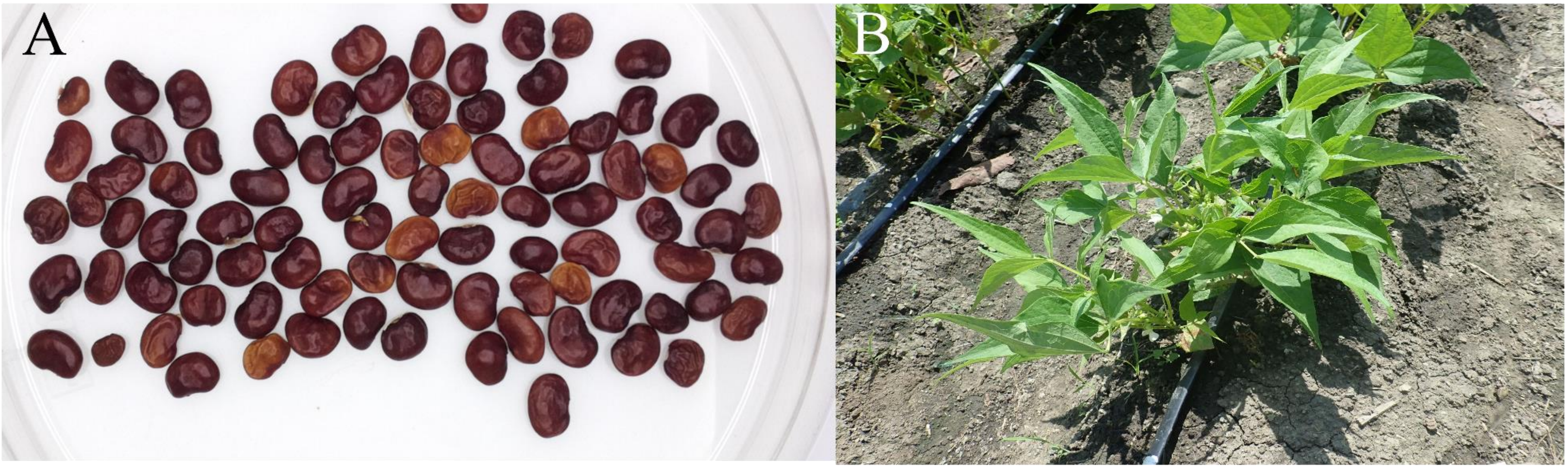

Supplementary Figure 4: Phenotypic traits characteristic of wild tepary bean present in F5:6 IMAWT lines. **A.** GCDT174 exhibited angular and wrinkled seeds. **B.** GCDT269 exhibited lanceolate leaves distinctive of tepary bean.

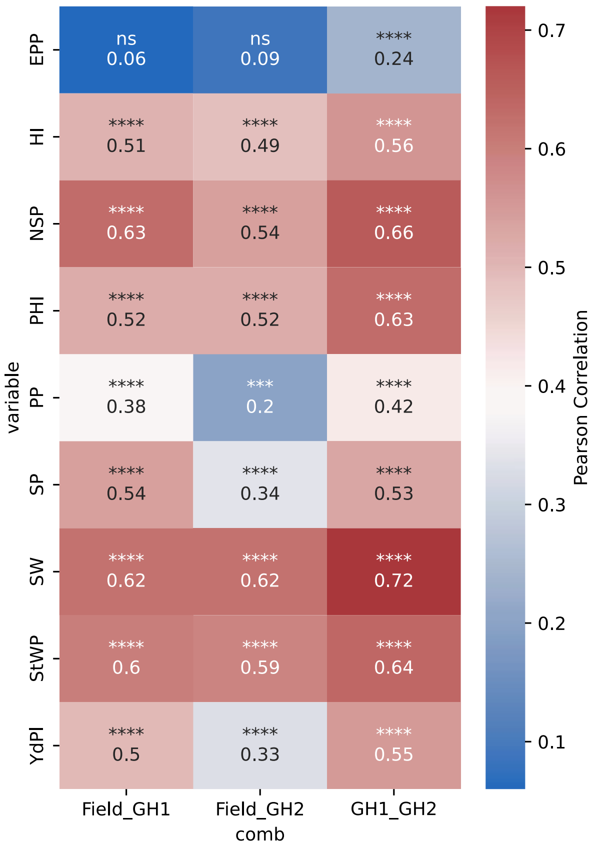

Supplementary Figure 5: Correlation between environments using Pearson correlation coefficient. Asterisks indicate the significance of Pearson correlation coefficients ns P > 0.05; * P ≤ 0.05; ** P ≤ 0.01; *** P ≤ 0.001; **** P ≤ 0.0001. **EPP**: Empty pods per plant (pods/plant). **NSP**: Number of seeds per pod (seeds/pod). **PHI:** Pod harvest index (%). **PP**: Pods per plant (pods/plant). **SP**: Seeds per plant (seeds/plant). **SW**: Seed weight (g/100 seeds). **StWP**: Dry stem weight per plant (g/plant). **YdPl**: Yield per plant (g/plant). **HI**: Harvest index (%).

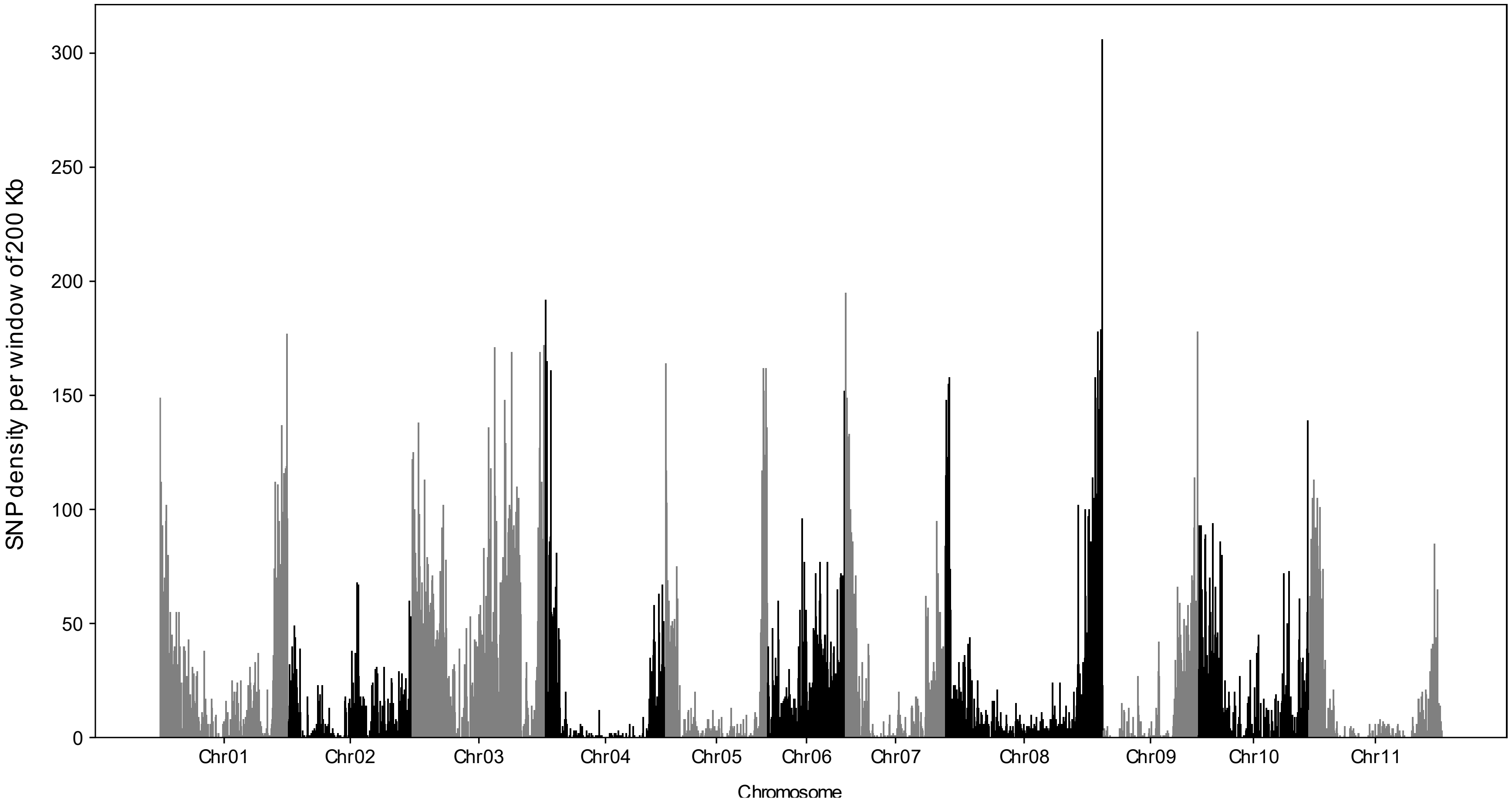

Supplementary Figure 6: Genome SNP density in fixed windows of 100 Kb for IMAWT population genotypic matrix composed by 24.205 SNPs.

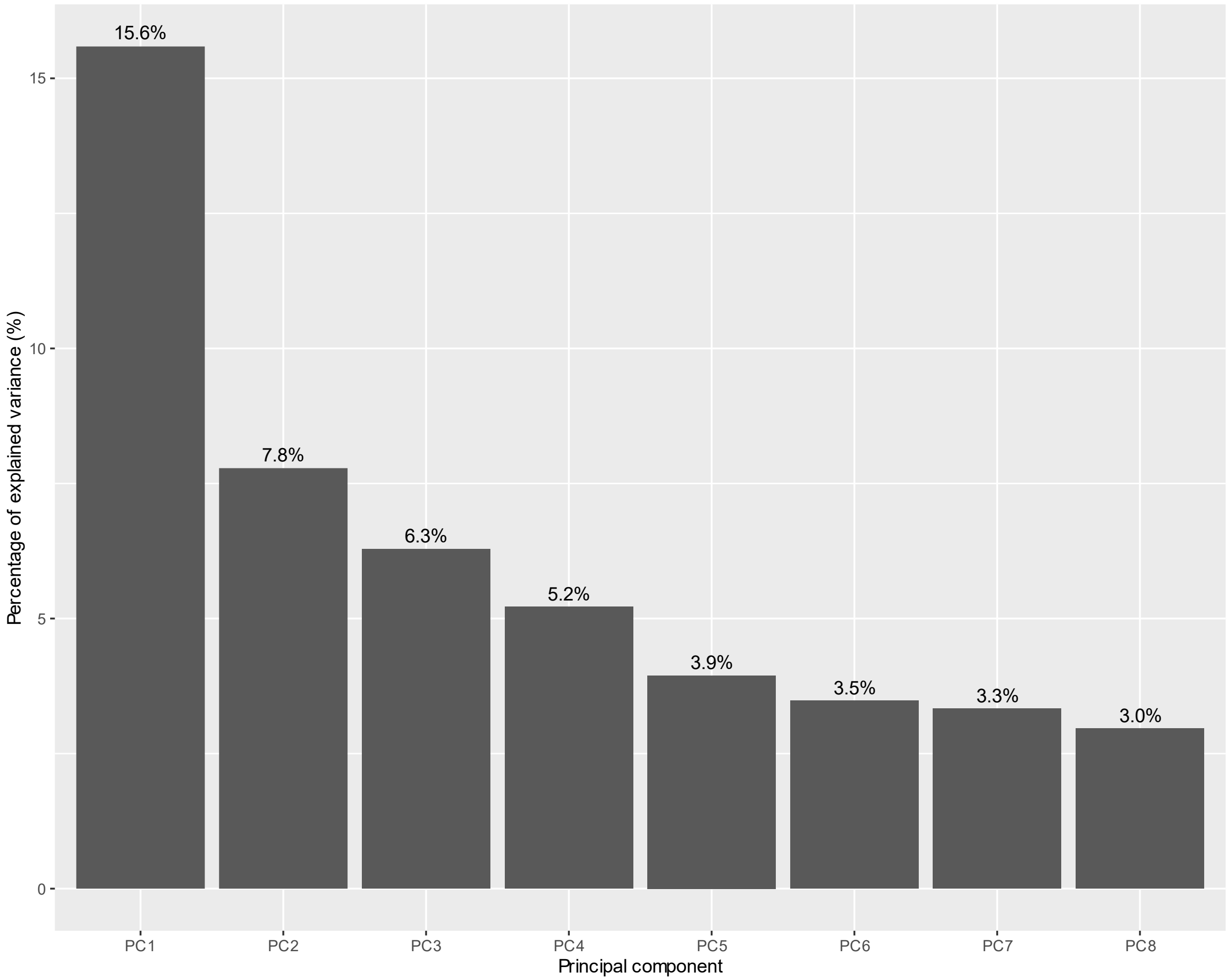

Supplementary Figure 7: Principal component analysis of 24.205 bi-allelic SNPs. Explained variance by the first eight principal components.
