# Supplementary material for: Interspecific common bean population derived from Phaseolus acutifolius using a bridging genotype demonstrate useful adaptation to heat tolerance
Source: Front Plant Sci. 2023 May 12;14:1145858. doi: 10.3389/fpls.2023.1145858 (PMC10246688; doi:10.3389/fpls.2023.1145858)
Supplement: Supplementary file 1 [file DataSheet_1.zip › Image 2.pdf]

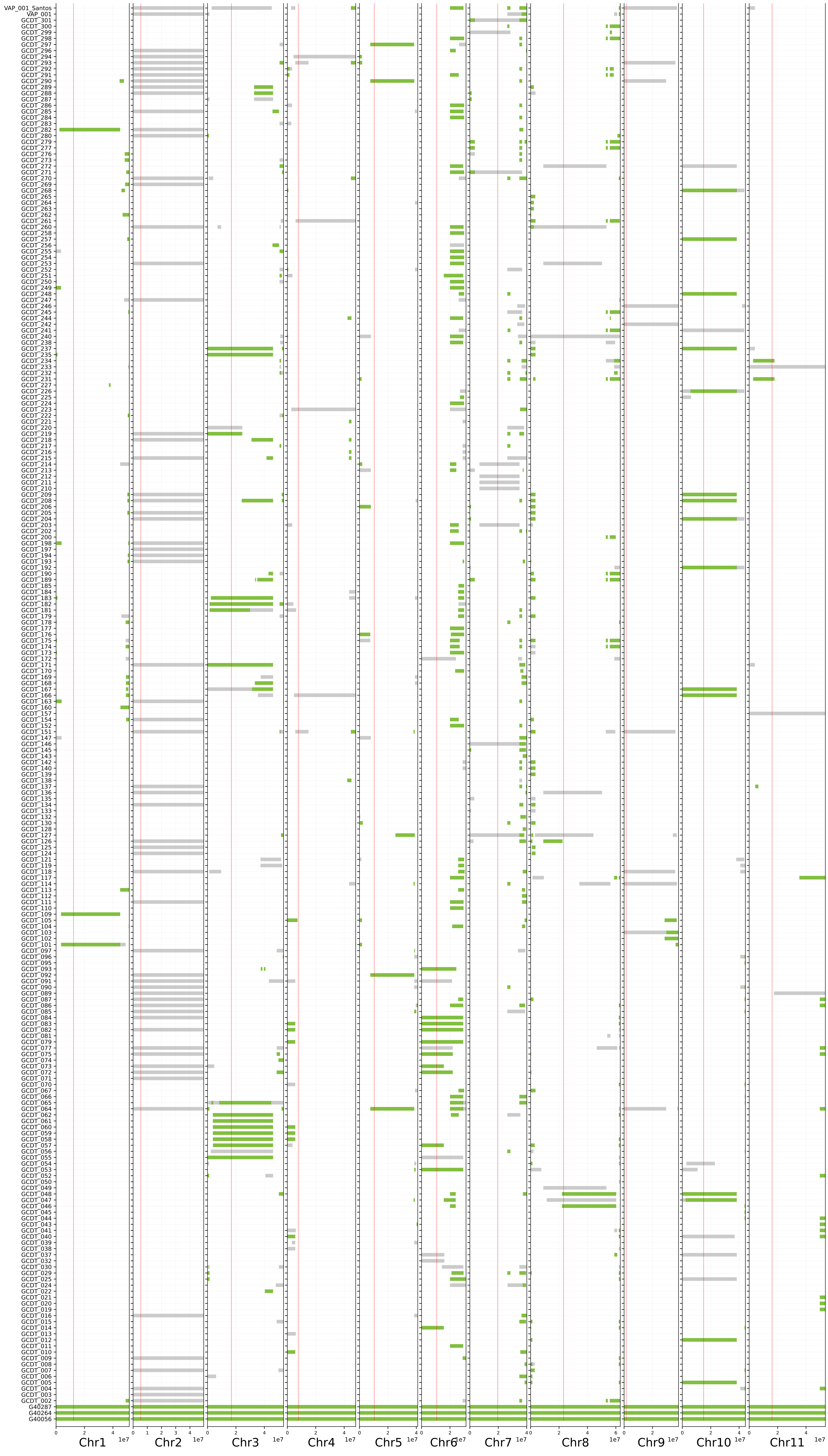

Supplementary Figure 8: *Acutifolii* Introgressions detected in the IMAWT population with a subset of contrasting biallelic SNPs between *Acutifolii* samples and common bean parental lines. Only samples with at least one introgression were plotted for a total of 203 interspecific $F_{5:6}$ families and a total of 465 introgression events. Tepary parental lines were included in the bottom for reference. The red line indicates according centromere ubication to v2.1 reference genome (Schmutz *et al* ., 2014)
