# Supplementary material for: Interspecific common bean population derived from Phaseolus acutifolius using a bridging genotype demonstrate useful adaptation to heat tolerance
Source: Front Plant Sci. 2023 May 12;14:1145858. doi: 10.3389/fpls.2023.1145858 (PMC10246688; doi:10.3389/fpls.2023.1145858)
Supplement: Supplementary file 1 [file DataSheet_1.zip › Image 3.PDF]

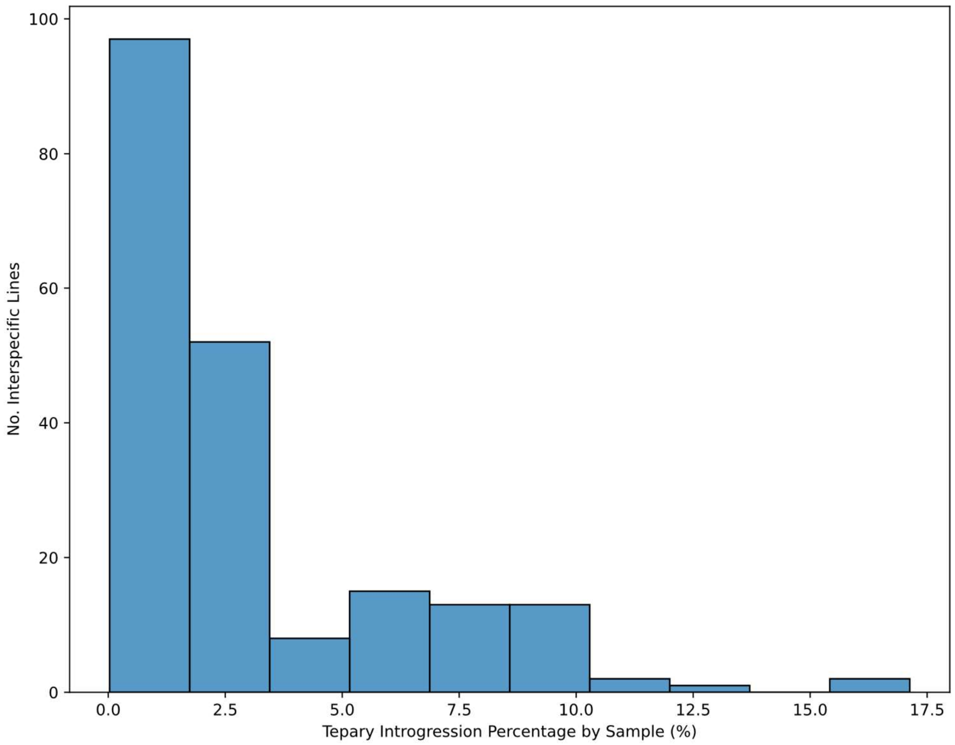

Supplementary Figure 9: Introgression percentage by sample among lines that carries at least one introgression event. Minimum introgression percentage was 0.03% and maximum 17.13%.
