# Supplementary material for: Interspecific common bean population derived from Phaseolus acutifolius using a bridging genotype demonstrate useful adaptation to heat tolerance
Source: Front Plant Sci. 2023 May 12;14:1145858. doi: 10.3389/fpls.2023.1145858 (PMC10246688; doi:10.3389/fpls.2023.1145858)
Supplement: Supplementary file 1 [file DataSheet_1.zip › Image 4.PDF]

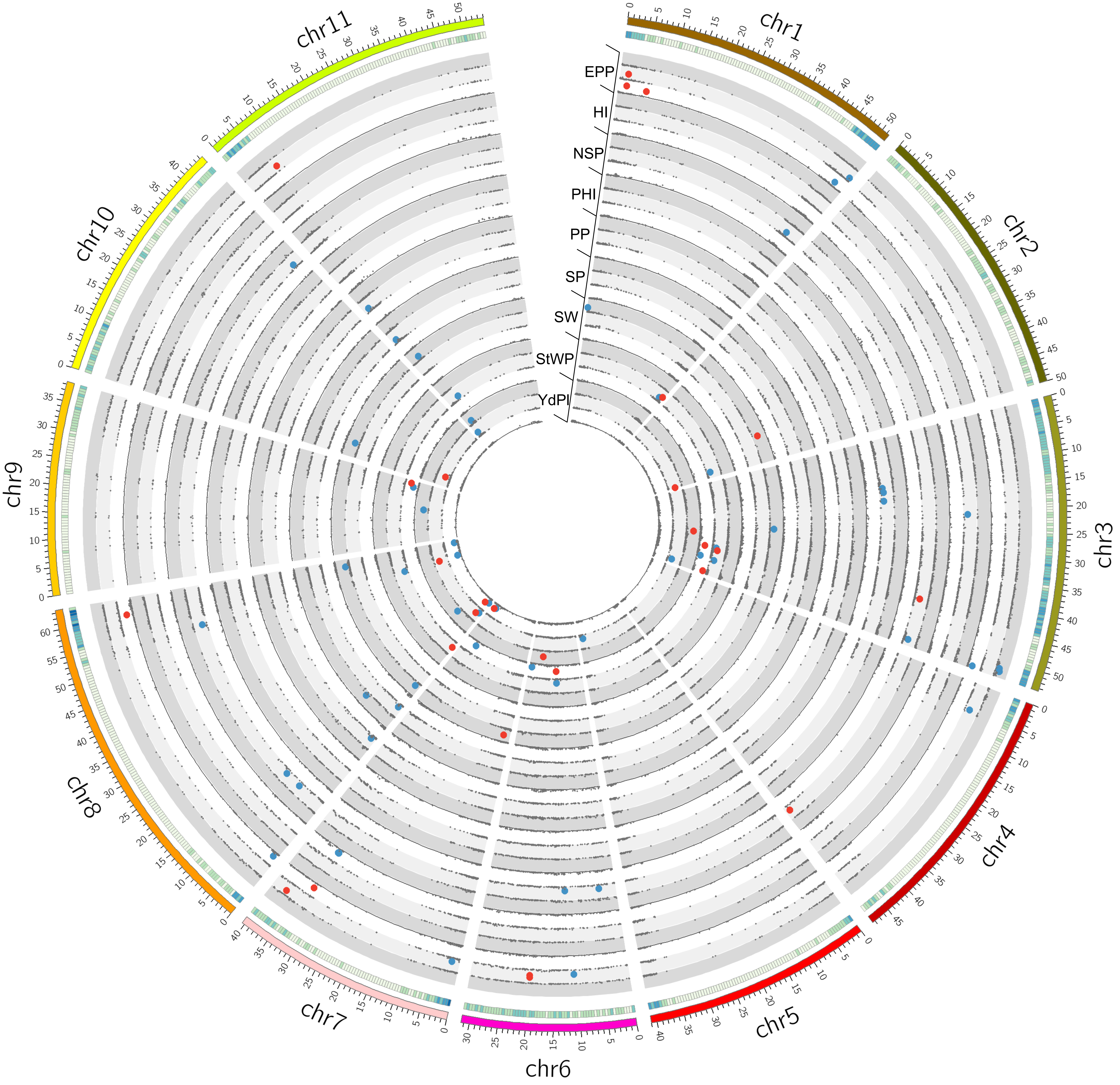

Supplementary Figure 10: Manhattan plots for evaluated traits in three environments. The outermost ring indicates chromosome number followed by variant density in 500 kb windows of genotype matrix used for GWAS analysis. The next rings are grouped by trait, the darker ring represent manhattan plot in NS environment whereas gray and white rings in GH1 and GH2, respectively. Red markers are the SNPs that exceed the Bonferroni significance threshold ($-\log_{10}$(P-value) > 5.7) whereas the blue markers are SNPs with high probability but below Bonferroni threshold ($-\log_{10}$(P-value) > 4)
