# Supplementary material for: Interspecific common bean population derived from Phaseolus acutifolius using a bridging genotype demonstrate useful adaptation to heat tolerance
Source: Front Plant Sci. 2023 May 12;14:1145858. doi: 10.3389/fpls.2023.1145858 (PMC10246688; doi:10.3389/fpls.2023.1145858)
Supplement: Supplementary file 1 [file DataSheet_1.zip › Image 5.pdf]

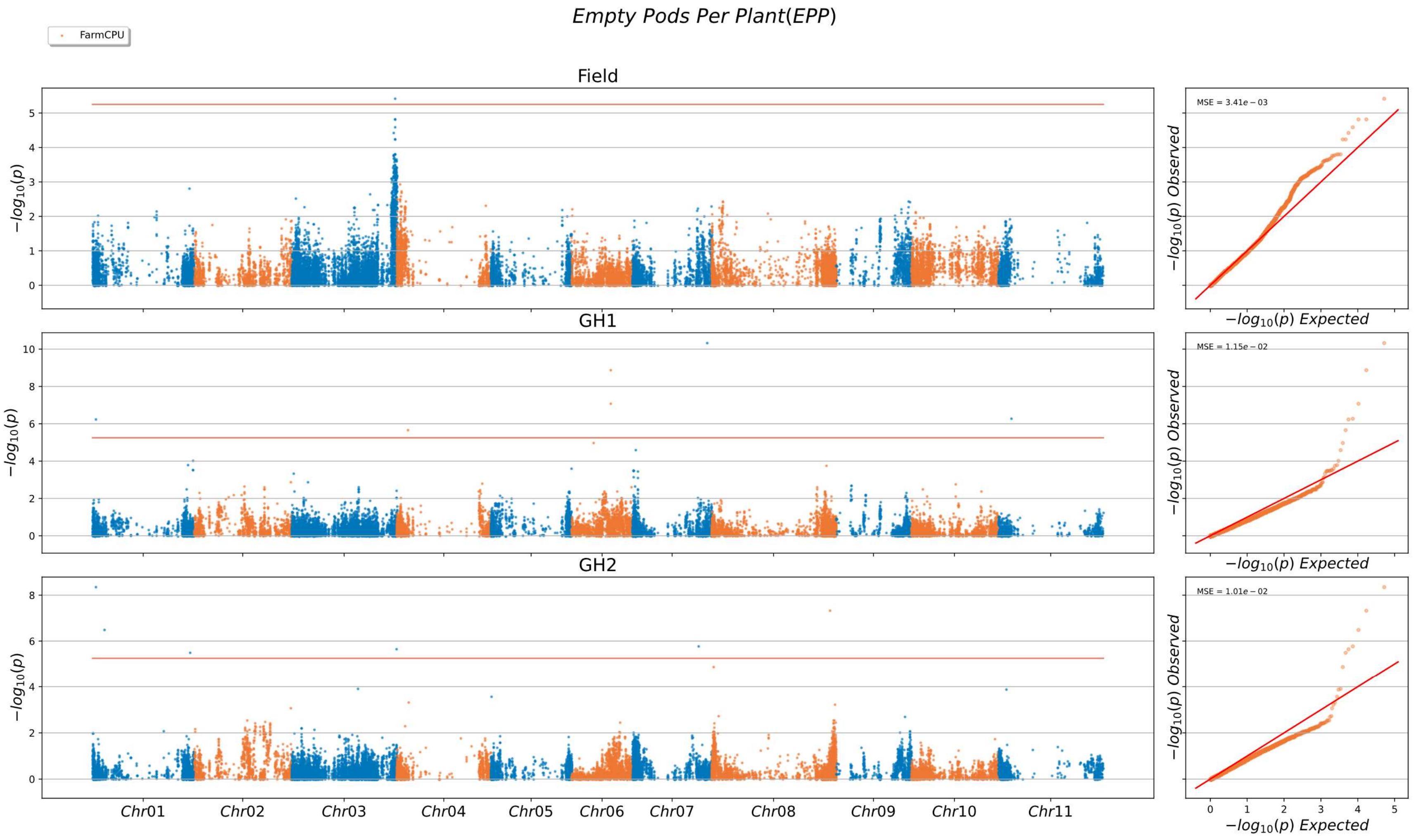

Supplementary Figure 11: Manhattan plots using FarmCPU model for Empty Pods per Plant (EPP) in the three environments evaluated. Left Manhattan plot. Bonferronni threshold red line for an α=0.05 at -$\log_{10}$(P-value) = 5.7. Right. QQ plot, MSE (mean square error) was calculated as a measure of model fit.

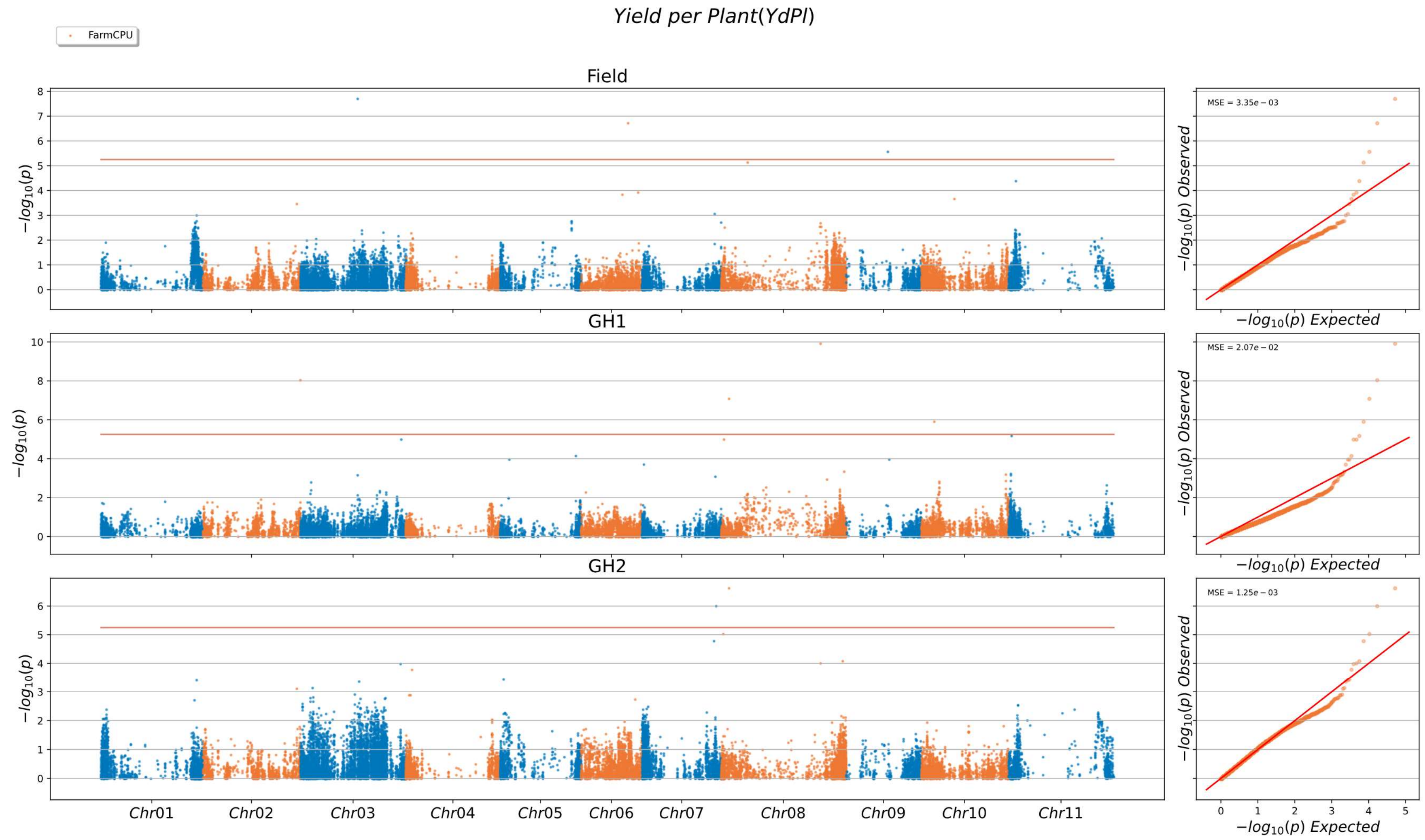

Supplementary Figure 12: Manhattan plots using FarmCPU model for Yield per Plant (YdPl) in the three environments evaluated. Left Manhattan plot. Bonferronni threshold red line for an α=0.05 at -$\log_{10}$(P-value) = 5.7. Right. QQ plot, MSE (mean square error) was calculated as a measure of model fit.

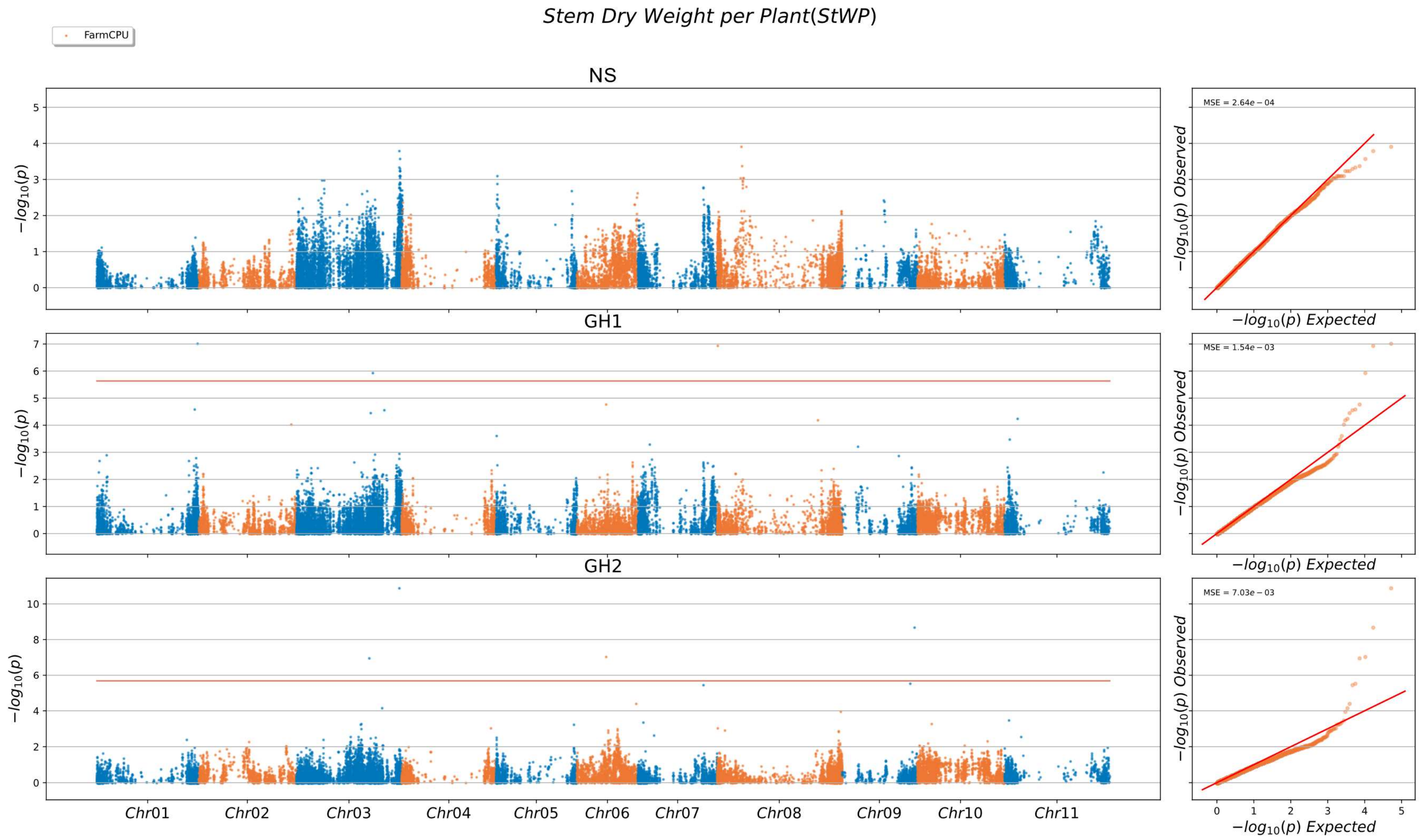

Supplementary Figure 13: Manhattan plots using FarmCPU model for dry weight of leafless stems per plot (StWP) in the three environments evaluated. Left Manhattan plot. Bonferronni threshold red line for an α=0.05 at -$\log_{10}$(P-value) = 5.7. Right. QQ plot, MSE (mean square error) was calculated as a measure of model fit.

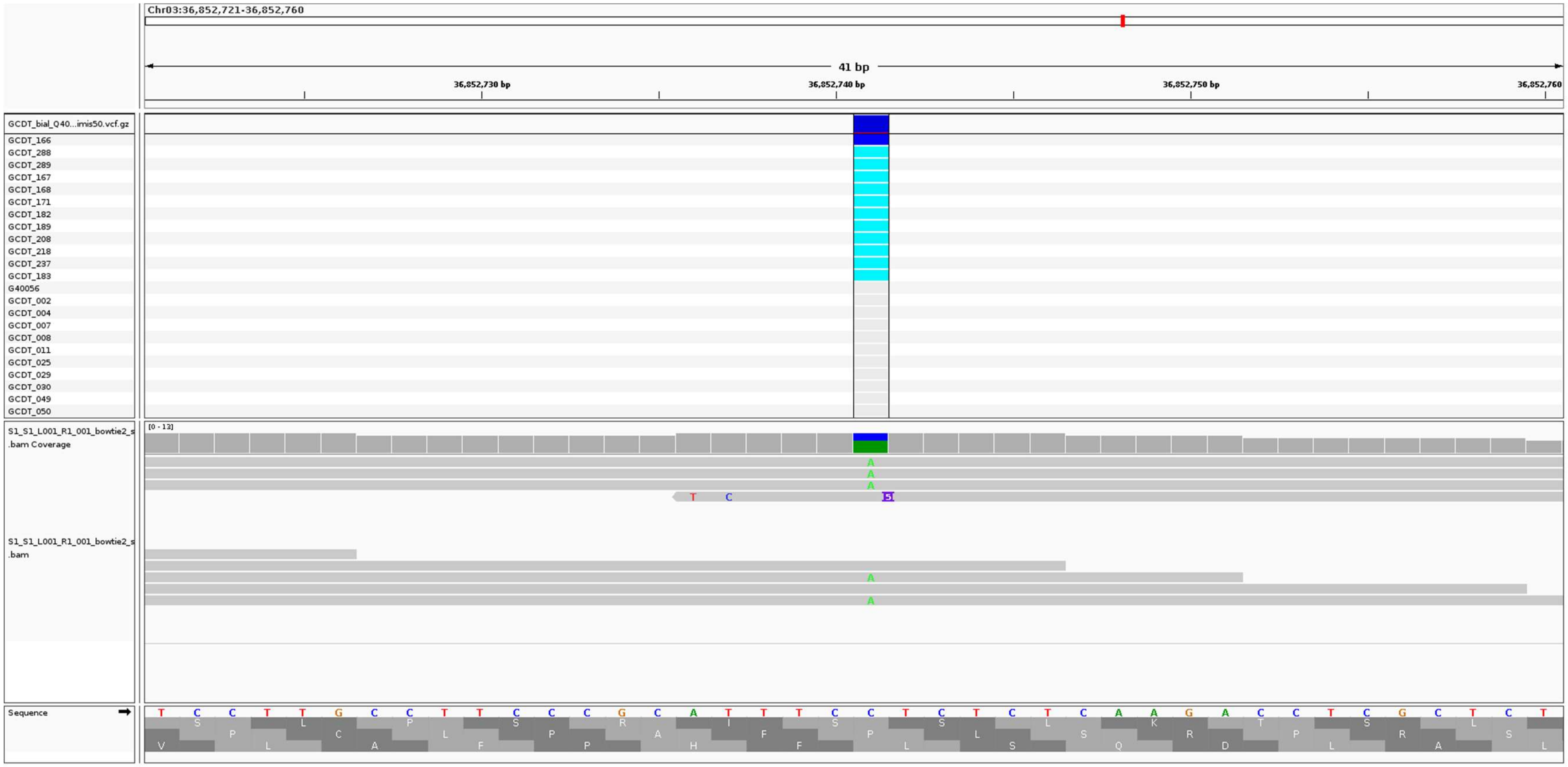

Supplementary Figure 14: VAP 1 (lower track) seems to be the putative source of alternative allele in the QTN StWP3.2. Information took from Barrera *et al.,* (2022).

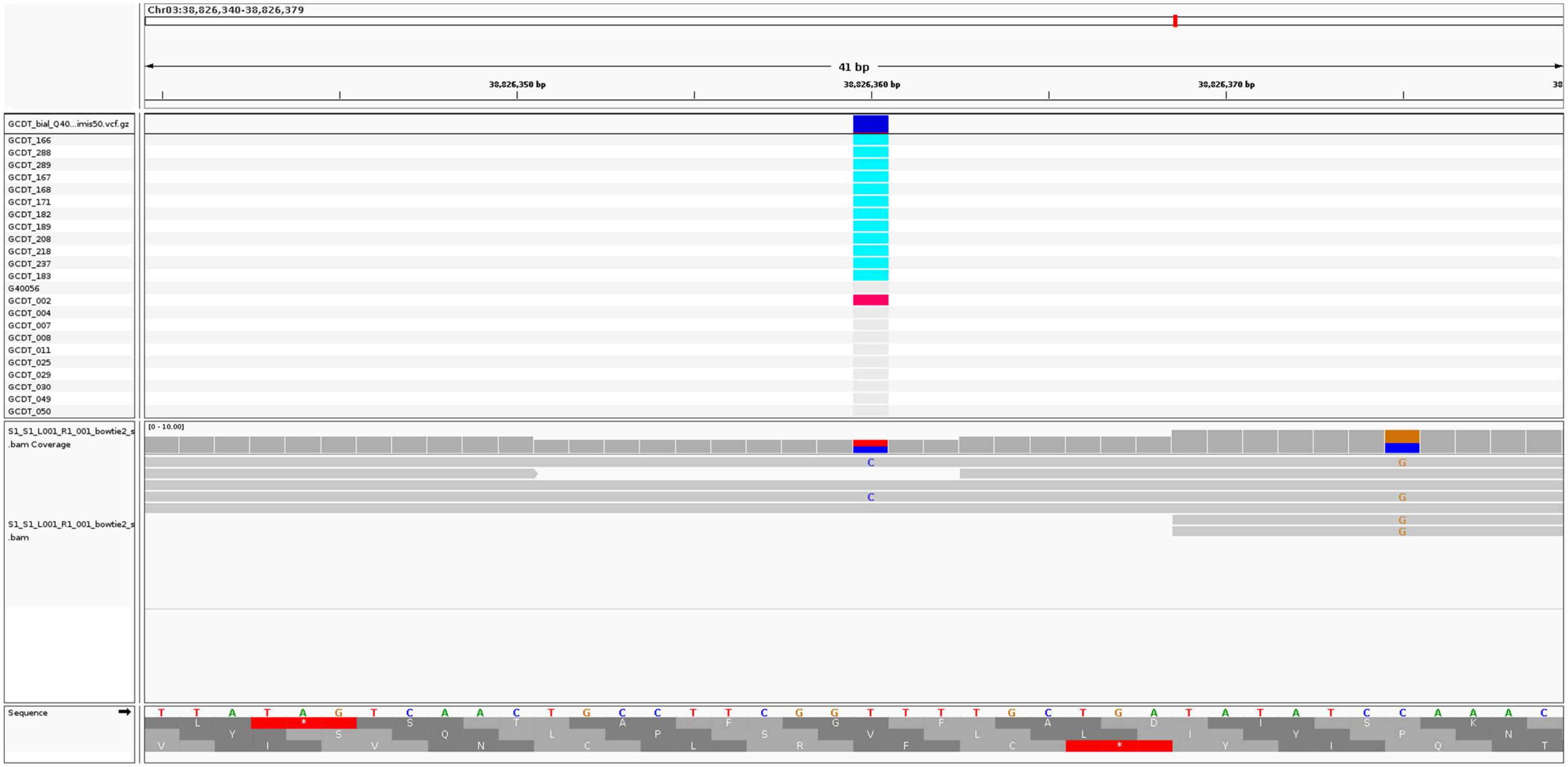

Supplementary Figure 15: VAP 1 (lower track) seems to be the putative source of alternative allele in the QTN StWP3.3. Information took from Barrera *et al.,* (2022).

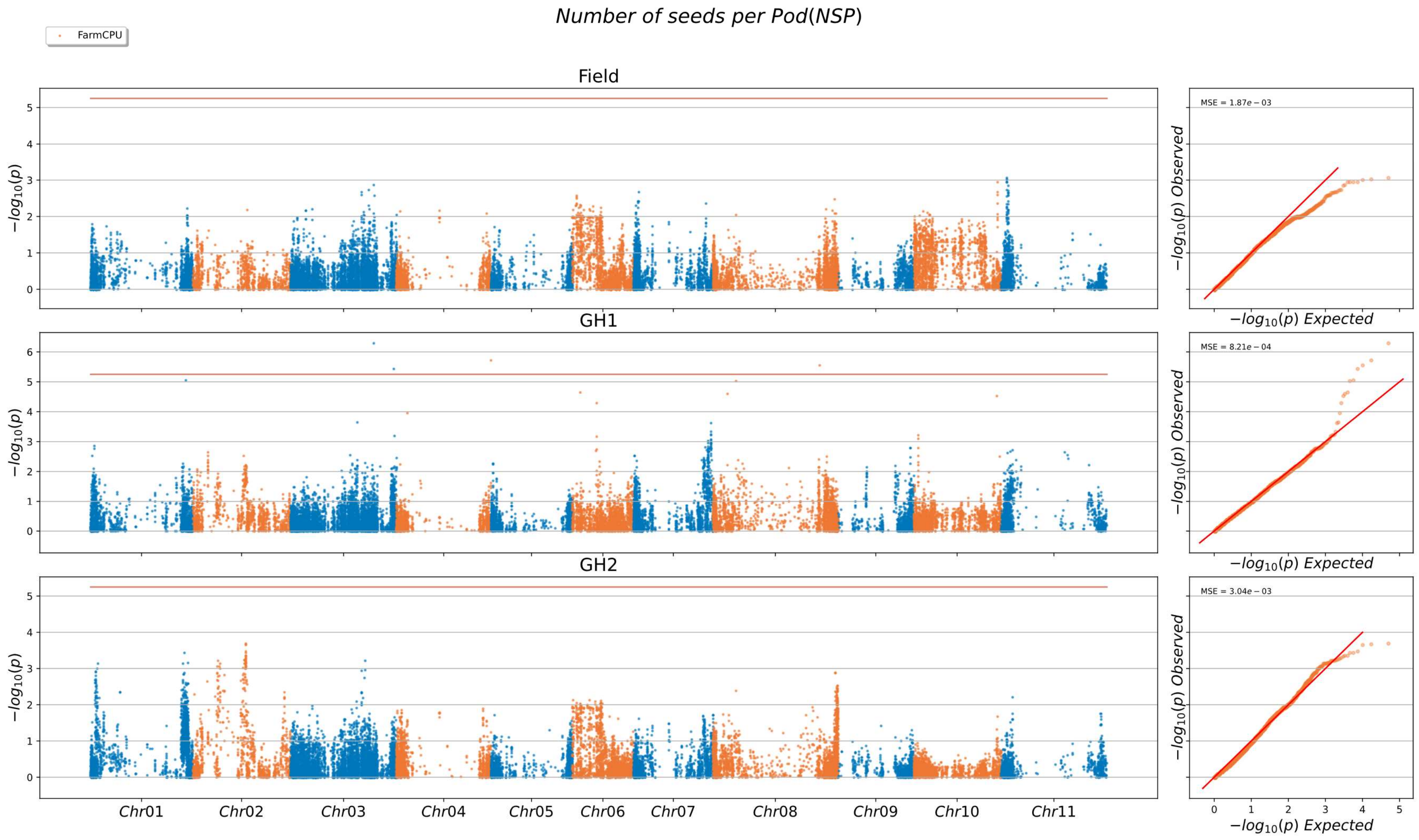

Supplementary Figure 16: Manhattan plots using FarmCPU model Number of Seeds per pod (NSP) in the three environments evaluated. Left Manhattan plot. Bonferronni threshold red line for an α=0.05 at -log_{10}(P-value) = 5.7. Right. QQ plot, MSE (mean square error) was calculated as a measure of model fit.
