# Supplementary material for: Interspecific common bean population derived from Phaseolus acutifolius using a bridging genotype demonstrate useful adaptation to heat tolerance
Source: Front Plant Sci. 2023 May 12;14:1145858. doi: 10.3389/fpls.2023.1145858 (PMC10246688; doi:10.3389/fpls.2023.1145858)
Supplement: Supplementary file 1 [file DataSheet_1.zip › Table 2.PDF]

Supplementary Table 2: Wide sense heritabilities for each trait and environment tested in this study. We used the method for computing herit ability proposed by *(Cullis* et al., *2006)*.

Trait	varG	varE	H^2	r^2	cv	Location
EPP	0.095	0.205	0.34	0.546	58.44	Field
EPP	2.58	2.712	0.51	0.716	64.65	GH1
EPP	2.767	0.757	0.79	0.932	32.06	GH2
NSP	0.412	0.261	0.64	0.823	10.64	Field
NSP	0.471	0.176	**0.75**	0.904	9.17	GH1
NSP	0.362	0.197	0.67	0.859	9.96	GH2
PHI	26.705	15.511	0.66	0.831	3.63	Field
PHI	56.36	16.378	0.79	0.922	3.31	GH1
PHI	48.995	12.003	0.82	0.937	2.67	GH2
PP	2.89	3.851	0.46	0.729	22.13	Field
PP	8.945	8.712	0.53	0.776	24.3	GH1
PP	5.671	5.237	0.54	0.777	22.41	GH2
SP	51.086	54.173	0.52	0.76	24.17	Field
SP	122.632	73.149	**0.64**	0.853	22.21	GH1
SP	65.393	39.584	0.63	0.853	19.61	GH2
StWP	0.911	0.414	0.71	0.888	20.47	Field
StWP	4.179	5.246	0.48	0.692	39.17	GH1
StWP	3.358	0.861	0.81	0.933	15.75	GH2
SW	16.668	4.564	0.8	0.928	5.97	Field
SW	16.613	6.75	0.74	0.887	7.56	GH1
SW	18.557	5.519	0.79	0.92	6.17	GH2
YdPl	7.461	6.584	0.56	0.764	25.58	Field
YdPl	9.339	5.174	0.66	0.857	23.35	GH1
YdPl	5.958	2.854	0.67	0.902	17.94	GH2
HI	23.309	16.998	0.62	0.79	3.43	Field
HI	129.771	46.226	**0.76**	0.903	5.36	GH1
HI	89.723	31.932	0.75	0.908	4.23	GH2

EPP: Empty pods per plant (pods/plant). **NSP**: Number of seeds per pod (seeds/pod). **PHI:** Pod harvest index (%). **PP**: Pods per plant (pods/plant). **SP**: Seeds per plant (seeds/plant). **SW**: Seed weight (g/100 seeds). **StWP**: Dry stem weight per plant (g/plant). **YdPl**: Yield per plant (g/plant). **HI**: Harvest index (%).
