# Supplementary material for: Interspecific common bean population derived from Phaseolus acutifolius using a bridging genotype demonstrate useful adaptation to heat tolerance
Source: Front Plant Sci. 2023 May 12;14:1145858. doi: 10.3389/fpls.2023.1145858 (PMC10246688; doi:10.3389/fpls.2023.1145858)
Supplement: Supplementary file 1 [file DataSheet_1.zip › Table 3.PDF]

Supplementary Table 3: Yield per plant (YdPl) by each parental line in three environments. NS: Non-stress, GH1 and GH2: Heat stress greenhouses (HS).

Genotype	YdPl NS		YdPl GH1		YdPl GH2	
**G40287**	8.35	A	14.89	A	18.14	A
**G40056**	8.02	A	13.92	A	10.61	B
**SEN 118**	11.81	A	6.65	B	9.46	B
**SEF 10**	9.16	A	4.64	B	6.95	C
**SMR 155**	10.40	A	5.44	B	6.83	C
**ICTA LIGERO**	6.32	B	4.49	B	6.55	C
**SMC 214**	7.50	A	7.04	B	6.52	C
**VAP 001**	10.43	A	6.16	B	5.84	D
**LSD**	5.03		4.46		3.31	

Contrasting groups where obtained with least significant difference (LSD) with an alpha of 0.05 using the formula: $LSD = 1.96 \cdot \sqrt{MSE \cdot \left(\frac{1}{rep\ 1} + \frac{1}{rep\ 2}\right)}$.
