# Supplementary material for: Interspecific common bean population derived from Phaseolus acutifolius using a bridging genotype demonstrate useful adaptation to heat tolerance
Source: Front Plant Sci. 2023 May 12;14:1145858. doi: 10.3389/fpls.2023.1145858 (PMC10246688; doi:10.3389/fpls.2023.1145858)
Supplement: Supplementary file 1 [file DataSheet_1.zip › Table 4.PDF]

Supplementary Table 4: Summary of introgression analysis. No. SNPs represent the number of contrasting variants between wild tepary and common bean parental lines.

Chromosome	No. SNPs	Min length (bp)	Max length (bp)	Pop. Cov	Introgression Events
Chr01	1219	383,589	42,362,984	100.00%	37
Chr02	17	-	-	-	-
Chr03	2807	255,898	45,934,831	100.00%	57
Chr04	361	455,674	6,959,765	26.47%	22
Chr05	566	150,920	30,554,346	98.54%	22
Chr06	396	663,203	29,119,916	100.00%	74
Chr07	587	255,122	5,056,125	25.31%	95
Chr08	1106	165,964	37,801,033	90.65%	109
Chr09	123	1,842,301	9,585,576	25.03%	4
Chr10	655	350,668	38,124,365	86.67%	27
Chr11	78	1,789,416	18,100,871	61.06%	18

Min and Max length: Maximum and minimum size of detected introgression fragment among IMAWT population. **Pop. Cov:** Coverage of common bean genome with introgressions of wild tepary using as reference the common bean genome v2.1 (Schmutz et al., 2014). **Introgression events**: Number of introgressions detected among IMAWT population.
